# Racial and Ethnic Differences in Prostate Cancer Epidemiology Across Disease States in the VA

**DOI:** 10.1001/jamanetworkopen.2024.45505

**Published:** 2024-11-15

**Authors:** Shannon R. Stock, Michael T. Burns, Justin Waller, Amanda M. De Hoedt, Joshua A. Parrish, Sameer Ghate, Jeri Kim, Irene M. Shui, Stephen J. Freedland

**Affiliations:** 1 Department of Surgery, Durham VA Health Care System, Durham, North Carolina; 2 Department of Mathematics and Computer Science, College of the Holy Cross, Worcester, Massachusetts; 3 Merck & Co, Inc, Kenilworth, New Jersey; 4 Department of Urology, Cedars-Sinai Medical Center, Los Angeles, California

## Abstract

**Importance:**

Prostate cancer (PC) care has evolved rapidly as a result of changes in prostate-specific antigen testing, novel imaging, and newer treatments. The impact of these changes on PC epidemiology and racial disparities across disease states remains underexplored.

**Objective:**

To characterize racial and ethnic differences in the epidemiology of PC states, including nonmetastatic hormone-sensitive PC (nmHSPC), metastatic HSPC (mHSPC), nonmetastatic castration-resistant PC (nmCRPC), and metastatic CRPC (mCRPC).

**Design, Setting, and Participants:**

This is a retrospective, population-based cohort study of male US veterans aged 40 years and older with known race and ethnicity and no non-PC malignant neoplasm before study entry receiving care through the Veterans Health Administration. The study period was from 2012 to 2020, with follow-up through 2021. To identify active users, data capture included visits 18 months before and after the study period. Data analysis was performed from March to August 2023.

**Exposure:**

Self-identified race and ethnicity, classified as Black, White, or Hispanic.

**Main Outcomes and Measures:**

The primary outcomes were annual age-adjusted incidence rates (IRs) and point prevalence for PC states by race and ethnicity. Trends were evaluated using joinpoint regression. Time to disease progression or death was estimated using nonparametric cumulative incidence. Competing risk models adjusted for age assessed the association of race and ethnicity on disease progression.

**Results:**

The study included 6 539 001 veterans (median [IQR] age, 65 [56-74] years), of whom 476 227 had PC (median [IQR] age, 69 [63-75] years). IRs varied by time frame and disease state. Across all states and years, the relative risk among Black vs White patients ranged from 2.09 (95% CI, 2.01-2.18; *P* < .001) for nmHSPC in 2012 to 4.12 (95% CI, 3.39-5.02; *P* < .001) for nmCRPC in 2017. In nmHSPC, hazard ratios for progression to mHSPC and nmCRPC were 1.36 (95% CI, 1.33-1.40) and 1.60 (95% CI, 1.51-1.70), respectively, for Black patients and 1.38 (95% CI, 1.31-1.45) and 1.55 (95% CI, 1.40-1.72), respectively, for Hispanic patients vs White patients. In contrast, in mCRPC, the hazard ratio for death was lower for Black (0.84; 95% CI, 0.81-0.88) and Hispanic (0.76; 95% CI, 0.69-0.83) patients compared with White patients.

**Conclusions and Relevance:**

This cohort study of veterans found that Black patients had more than 2-fold higher incidence of all disease states vs White patients. Progression risk was higher for Black and Hispanic patients in early-stage disease, but lower in later disease stages. Despite equal access, Black patients disproportionately experience PC, although progression risks relative to White patients differed according to disease state.

## Key Points

**Question:**

Are there racial and ethnic differences in the epidemiology and progression of prostate cancer (PC) across disease states?

**Findings:**

In this cohort study of more than 6 million US veterans, Black patients had a greater than 2-fold higher risk of PC vs White patients across all disease states. Disease progression risk varied by disease state and race and ethnicity, with Black and Hispanic patients having higher progression risks in early states but lower risks in later states.

**Meaning:**

Despite equal access, racial and ethnic disparities persist in PC incidence and progression, highlighting the importance of evaluating these differences across the disease continuum to develop targeted strategies and improve outcomes.

## Teaser

This cohort study examines the racial and ethnic differences in the epidemiology of prostate cancer states among US veterans treated at Veterans Affairs (VA) facilities.

## Introduction

The landscape of prostate cancer (PC) screening and detection has changed remarkably over the past 15 years. Although the 2012 US Preventive Services Task Force guidelines recommended against prostate-specific antigen (PSA) screening, this was reversed in 2018, coinciding with shifts in PSA testing practices.^1,2^ In addition, the adoption of novel positron emission tomography imaging has enabled earlier detection of metastatic disease than conventional bone and computerized tomography scans.^2,3^

For individuals whose cancer progresses beyond localized, nonmetastatic hormone-sensitive PC (nmHSPC), some will receive a diagnosis of and be treated for metastatic hormone-sensitive PC (mHSPC), whereas others will progress to nonmetastatic castration-resistant PC (nmCRPC) following androgen deprivation therapy (ADT). Nearly all patients who die from PC will have metastatic CRPC (mCRPC) before death.^4^ The past 15 years have also seen major changes in the treatment of advanced PC; cutting-edge therapies introduced earlier in the disease have demonstrated substantial survival benefits.^5,6,7,8,9,10^

Given these breakthroughs, there is a need to characterize the evolving epidemiology and disease progression rates across PC disease states defined by metastases and castration resistance, and to explore racial and ethnic disparities. This information will help us better understand how disease burden is evolving, and is vital to defining target populations and follow-up durations for end-point analysis in future trials. It is well known that there are substantial disparities associated with Black race for PC incidence and newly diagnosed metastatic PC.^11,12,13^ However, there are limited studies that have accounted for castration resistance, a critical factor given that CRPC marks a pivotal point in clinical decision-making and prognosis. Furthermore, considering PC’s typically slow progression, except for mCRPC, contemporary data on disease progression rates from trials rely on relatively short follow-up, and there is a scarcity of recent natural history studies. Moreover, trials often enroll very specific patient populations (ie, healthier and less diverse populations) and, thus, may not fully reflect natural history in the general population.

The Veterans Health Administration (VHA), the largest integrated health care system in the US, offers an exceptional infrastructure for population-based studies owing to its provision of equal access and a diverse population.^14^ Leveraging the VHA, we conducted a comprehensive longitudinal cohort study of PC epidemiology from 2012 to 2021, covering the disease continuum, from newly diagnosed PC (nmHSPC and de novo mHSPC) to disease states resulting from progression (recurrent mHSPC, nmCRPC, and mCRPC). Importantly, we also examined racial and ethnic differences within these measures. Here, we provide incidence, prevalence, and disease transition rates over time and by race and ethnicity.

## Methods

### Study Design and Patient Population

We conducted a retrospective cohort study of patients of male sex aged 40 years or older who were active VHA users, defined as individuals with 2 or more visits to VHA centers within any 18-month interval during the study period of January 1, 2012, to December 31, 2020, with follow-up for outcomes through December 31, 2021. To determine active user status, data capture included visits 18 months before and after the study. Patients who stopped being active users were considered lost to follow-up and were censored 6 months after their last visit. Given this open cohort design, patients could leave and reenter the cohort according to active user status, although such occurrences were rare.

We excluded patients with prior non-PC malignant tumor (except nonmelanoma skin cancer) before the study period. Patients in the base population who developed a non-PC malignant tumor, and patients with PC who developed a second primary malignant tumor after study start, were censored at the date of the non-PC diagnosis. This was done because claims-based *International Classification of Diseases, Ninth Revision (ICD-9)* or *International Statistical Classification of Diseases and Related Health Problems, Tenth Revision (ICD-10)* codes for metastases are not cancer-type specific, so censoring minimizes misclassification of metastatic PC when metastases may be attributed to non-PC malignant neoplasm. This study followed Strengthening the Reporting of Observational Studies in Epidemiology (STROBE) reporting guidelines with institutional review board approval from the Durham VHA. Data were collected with waivers of informed consent, in accordance with 45 CFR §46.

### Data Sources

We used inpatient, outpatient, and fee-basis claims data from the VHA’s Corporate Data Warehouse, the central repository for VHA electronic medical records, containing demographic, clinical, and radiology data. PC cases were identified by 2 or more *ICD-9* or *ICD-10* codes (*ICD-9*, 185; or *ICD-10*, C61) on different dates.

Race and ethnicity were self-reported, obtained from electronic health records, and categorized as non-Hispanic White (White), non-Hispanic Black (Black), Hispanic of any race (Hispanic), or other, which encompassed Alaska Native, American Indian, Asian, and Pacific Islander patients of non-Hispanic ethnicity. Age at diagnosis was categorized as 40 to 54, 55 to 64, 65 to 74, or greater than or equal to 75 years.

CRPC was identified using a structured query language algorithm incorporating *ICD-9* or *ICD-10* codes for CRPC, CRPC treatment, and searches for increasing PSA or metastatic disease development while receiving continuous ADT.^15^ We used 3 validated algorithms to detect metastatic disease: (1) *ICD-9* or *ICD-10* codes for metastases, (2) a novel algorithm combining *ICD-9* or *ICD-10* codes for metastases and common treatments for metastatic PC, and (3) a natural language processing algorithm that scans free-text radiology reports for evidence of metastases, which is highly accurate.^16^ The earliest metastatic disease date identified by any algorithm marked the metastatic PC diagnosis date. Patients with evidence of metastasis within 90 days of PC diagnosis were classified as having de novo mHSPC. The diagnosis date for each disease state (nmHSPC, de novo mHSPC, recurrent mHSPC, nmCRPC, or mCRPC) was the index date for the disease-specific cohort. Because patients could transition between different PC disease states, they were followed longitudinally and may have moved between cohorts.

### Statistical Analysis

Data analysis was performed from March to August 2023. Demographic, clinical, and comorbidity characteristics were summarized at the index date for each disease state. Annual age-adjusted incidence rates (IRs) and point prevalence per 100 000 person-years for each PC state were calculated by year and stratified by race and ethnicity. The base population for IRs included those at risk for each disease state (ie, disease-free active users and those with a less-advanced PC disease state). Person-time was censored upon incident diagnosis, disease progression, death, non-PC malignant neoplasm diagnosis, loss of active user status, or study end. Point prevalence was determined annually on December 31, according to the number of individuals in each PC disease state relative to the total study population.

Age-adjusted relative risks (RRs) were used to compare IRs and point prevalence across racial and ethnic groups. Joinpoint regression models were used to assess trends over time and identify significant change points. The estimated annual percentage change (APC) characterized temporal trends, with APC greater than 0 indicating rate increases and APC less than 0 indicating decreases. APCs not significantly different from 0 were termed steady. Owing to the impact of COVID-19 on PC diagnoses, 2020 data were excluded from the time trend analysis but included in eTables 1 to 16 in Supplement 1.^17,18^ IRs, point prevalence, and RRs were standardized using US Census age-specific, race-specific, and ethnicity-specific population estimates.^19^

We estimated 3-year, 5-year, and 10-year disease progression rates by disease state, stratified by race, using nonparametric estimates of cumulative incidence. Time zero was the index date for each disease state, and transitions to advanced states or death were competing risks. For nmHSPC, de novo mHSPC, recurrent mHSPC, and nmCRPC, age-adjusted estimates of the hazard ratio (HR) comparing risk of progression or death by race and ethnicity were obtained using Fine and Grey competing risk models. For mCRPC, a Cox proportional hazards model was used to adjust for age. Because time zero may have occurred before a patient’s study entry, all analyses accounted for left truncation.^20,21^

We conducted IR and prevalence analyses using SAS statistical software version 9.4 (SAS Institute), Joinpoint regression software version 4.9.0.0 (National Cancer Institute) was used for time trend analyses, and competing risk analyses were calculated using R statistical software version 4.1.0 (R Project for Statistical Computing).^22^ All statistical tests were 2-sided, with a significance level of α = .05.

## Results

**Figure 1.**
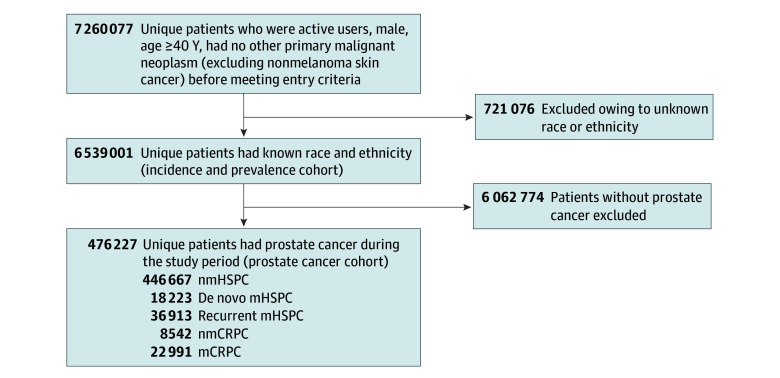
Study Enrollment Flowchart Patients who transitioned during the study period are counted in all relevant disease states. mCRPC indicates metastatic castrate-resistant prostate cancer; mHSPC, metastatic hormone–sensitive prostate cancer; nmCRPC, nonmetastatic castrate-resistant prostate cancer; nmHSPC, nonmetastatic hormone-sensitive prostate cancer.

Our cohort for estimating incidence and prevalence included 6 539 001 patients (Figure 1) with a median (IQR) age of 65 (56-74) years. Of these, 1 021 710 (16%) were Black, 379 478 were (6%) Hispanic, 4 904 324 were (75%) White, and 233 489 were (4%) other race. A total of 476 227 patients (median [IQR] age, 69 [63-75] years) received a diagnosis of PC before or during the study, forming our PC cohort. Table 1 details the PC cohort characteristics by disease state. eTables 1 to 5 in Supplement 1 provide these characteristics by race and ethnicity.

**Table 1.** Characteristics of the PC Cohort, Stratified by Disease State

Characteristic^a^	Participants, No. (%)				
	nmHSPC (n = 446 667)	De novo mHSPC (n = 18 223)	Recurrent mHSPC (n = 36 913)	nmCRPC (n = 8542)	mCRPC (n = 22 991)
Age at diagnosis of disease state, median (IQR), y	68.8 (63.2-75.0)	70.3 (64.2-78.3)	72.4 (67.0-79.4)	76.7 (69.7-84.5)	74.4 (68.3-82.6)
Race and ethnicity					
Hispanic	20 001 (4.5)	1025 (5.6)	2049 (5.6)	531 (6.2)	1144 (5.0)
Non-Hispanic Black	88 888 (19.9)	4929 (27.0)	9623 (26.1)	2317 (27.1)	5598 (24.3)
Non-Hispanic White	324 482 (72.6)	11 684 (64.1)	24 004 (65.0)	5364 (62.8)	15 538 (67.6)
Non-Hispanic other^b^	13 296 (3.0)	585 (3.2)	1237 (3.4)	330 (3.9)	711 (3.1)
US Census region at PC diagnosis					
Midwest	102 292 (22.9)	4054 (22.2)	8318 (22.5)	1933 (22.6)	5648 (24.6)
Northeast	71 469 (16.0)	2552 (14.0)	5332 (14.4)	1181 (13.8)	3394 (14.8)
Not in US	111 (<0.1)	4 (<0.1)	8 (<0.1)	NA	NA
Puerto Rico	7113 (1.6)	374 (2.1)	941 (2.5)	285 (3.3)	457 (2.0)
South	186 690 (41.8)	7565 (41.5)	15 185 (41.1)	3281 (38.4)	8643 (37.6)
West	78 992 (17.7)	3674 (20.2)	7129 (19.3)	1862 (21.8)	4849 (21.1)
Prostate-specific antigen at diagnosis of disease state, median (IQR), ng/mL	4.8 (0.5-7.9)	20.5 (7.0-110.3)	2.8 (0.2-13.3)	5.5 (3.2-11.5)	9.5 (3.1-37.9)
Missing, No.	216 151	6263	14 706	704	3947
Total Gleason score at PC diagnosis					
<6	265 (0.3)	7 (0.1)	39 (0.4)	4 (0.2)	14 (0.2)
6	30 486 (38.3)	586 (10.5)	3024 (30.4)	219 (11.6)	446 (7.4)
7 (3 + 4)	24 729 (31.1)	975 (17.4)	2708 (27.2)	340 (18.0)	771 (12.7)
7 (4 + 3)	10 495 (13.2)	715 (12.8)	1461 (14.7)	285 (15.1)	778 (12.9)
8	7144 (9.0)	1166 (20.8)	1261 (12.7)	369 (19.6)	1269 (21.0)
9-10	6385 (8.0)	2158 (38.5)	1445 (14.5)	670 (35.5)	2770 (45.8)
Missing, No.	367 163	12 616	26 975	6655	16 943
Site of metastasis at metastatic diagnosis					
Bone	NA	706 (56.9)	775 (51.1)	NA	812 (70.9)
Liver	NA	15 (1.2)	46 (3.0)	NA	5 (0.4)
Lung	NA	31 (2.5)	71 (4.7)	NA	17 (1.5)
Lymph	NA	177 (14.3)	319 (21.0)	NA	174 (15.2)
Multiple sites	NA	56 (4.5)	41 (2.7)	NA	51 (4.5)
Other or unspecified	NA	255 (20.6)	264 (17.4)	NA	87 (7.6)
Missing, No.	446 667	16 983	35 397	8542	21 845
Charlson Comorbidity Index at diagnosis of disease state, median (IQR)	0.0 (0.0-2.0)	1.0 (0.0-4.0)	3.0 (1.0-5.0)	2.0 (1.0-5.0)	4.0 (1.0-7.0)
Comorbidities at diagnosis of disease state					
Obesity	141 216 (37.9)	4846 (32.2)	10 296 (33.8)	2434 (40.0)	7131 (36.4)
Missing	74 306	3177	6432	2463	3395
Diabetes	112 025 (25.1)	6267 (34.4)	17 242 (46.7)	3829 (44.8)	9770 (42.5)
Dementia	4993 (1.1)	670 (3.7)	1895 (5.1)	514 (6.0)	1066 (4.6)
Cardiovascular disease	122 343 (27.4)	6859 (37.6)	19 160 (51.9)	4424 (51.8)	11 143 (48.5)
Hypertension	257 032 (57.5)	12 851 (70.5)	31 490 (85.3)	7313 (85.6)	18 674 (81.2)

Abbreviations: mCRPC, metastatic castrate-resistant prostate cancer; mHSPC, metastatic hormone–sensitive prostate cancer; NA, not applicable; nmCRPC, nonmetastatic castrate-resistant prostate cancer; nmHSPC, nonmetastatic hormone-sensitive prostate cancer; PC, prostate cancer.

SI conversion factor: To convert prostate-specific antigen to micrograms per liter, multiply by 1.

^a^ Patient characteristics were captured at the index date of each disease state and patients may have belonged to more than 1 disease state during the study period.

^b^ Other includes Alaska Native, American Indian, Asian, and Pacific Islander patients of non-Hispanic ethnicity.

### Incidence and Prevalence

**Figure 2.**
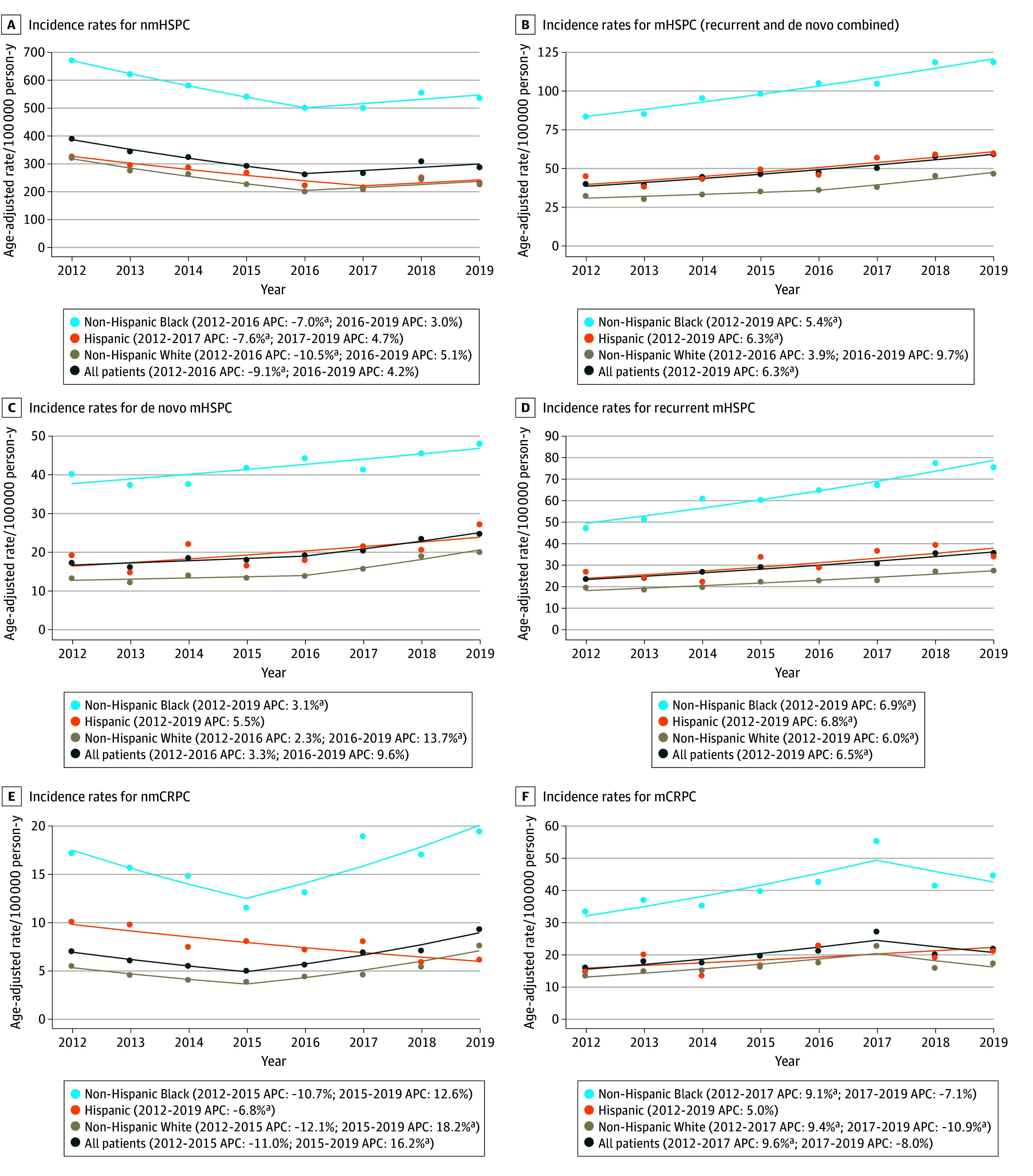
Age-Adjusted Incidence Rates (per 100 000 Person-Years) by Race, Ethnicity, Disease State, and Year The dots represent the observed rates; the solid line represents model-based estimates obtained by the joinpoint analysis, with the annual percentage change (APC) noted for each line segment. mCRPC indicates metastatic castrate-resistant prostate cancer; mHSPC, metastatic hormone–sensitive prostate cancer; nmCRPC, nonmetastatic castrate-resistant prostate cancer; and nmHSPC, nonmetastatic hormone-sensitive prostate cancer. ^a^Indicates that the APC is significantly different from 0 at α = .05.

Complete data on annual age-standardized IRs (per 100 000 person-years) by disease state for 2012 to 2020 are presented in eTables 6 and 7 in Supplement 1, which provide IR by race and ethnicity and RRs for Black vs White and Hispanic vs White patients. Time trend analyses are shown in Figure 2, with detailed APC information in eTables 8 and 9 in Supplement 1.

For nmHSPC, IRs decreased from 390 cases per 100 000 person-years (95% CI, 382-397 cases per 100 000 person-years) in 2012 to a nadir of 261 cases per 100 000 person-years (95% CI, 255-267 cases per 100 000 person-years) in 2016 (APC, −9.1%), and then remained steady through 2019. For mHSPC, including both de novo and recurrent disease, IRs increased from 40 cases per 100 000 person-years (95% CI, 38-42 cases per 100 000 person-years) in 2012 to 59 cases per 100 000 person-years (95% CI, 57-61 cases per 100 000 person-years) in 2019 (APC, 6.3%). These temporal trends were consistent by race and ethnicity.

IRs for nmCRPC remained steady from 2012 to 2015 and then increased from 5 cases per 100 000 person-years (95% CI, 4-5 cases per 100 000 person-years) in 2015 to 9 cases per 100 000 person-years (95% CI, 9-10 cases per 100 000 person-years) in 2019 (APC, 16.2%). For mCRPC, IRs increased from 16 cases per 100 000 person-years (95% CI, 15-17 cases per 100 000 person-years) in 2012 to 27 cases per 100 000 person-years (95% CI, 26-28 cases per 100 000 person-years) in 2017 (APC, 9.6%) and then remained steady through 2019. Trends for Black and White patients mirrored these patterns. However, for Hispanic patients, nmCRPC IRs declined throughout the study (APC, −6.8%), whereas mCRPC IRs remained steady.

The RR of PC was significantly higher for Black vs White patients across all disease states during the study, with RRs for nmHSPC between 2.09 (95% CI, 2.01-2.18; *P* < .001) and 2.51 (95% CI, 2.39-2.63; *P* < .001). In patients with mHSPC, nmCRPC, and mCRPC, RRs ranged from 2.33 (95% CI, 2.06-2.62; *P* < .001) to 4.12 (95% CI, 3.39-5.02; *P* < .001).

For Hispanic vs White patients, the RR of PC was elevated, but not always significant, across disease states. The largest RRs were observed in mHSPC and nmCRPC. For mHSPC, RRs were significant across the study and ranged from 1.26 (95% CI, 1.05-1.51; *P* = .01) to 1.50 (95% CI, 1.29-1.73; *P* < .001). For nmCRPC, RRs were significant between 2012 and 2017 and ranged from 1.64 (95% CI, 1.15-2.23; *P* = .006) to 2.15 (95% CI, 1.46-3.16; *P* < .001).

Annual age-standardized estimates of point prevalence are provided in eTables 10 and 11 in Supplement 1, and the time trend analysis is shown in the eFigure in Supplement 1. Across all disease states, trends in prevalence followed patterns similar to those for IRs. The magnitude of RR estimates for Black and Hispanic vs White patients was similar to IR estimates within nmHSPC and mHSPC but was higher in nmCRPC and mCRPC.

### Disease Progression Rates

**Figure 3.**
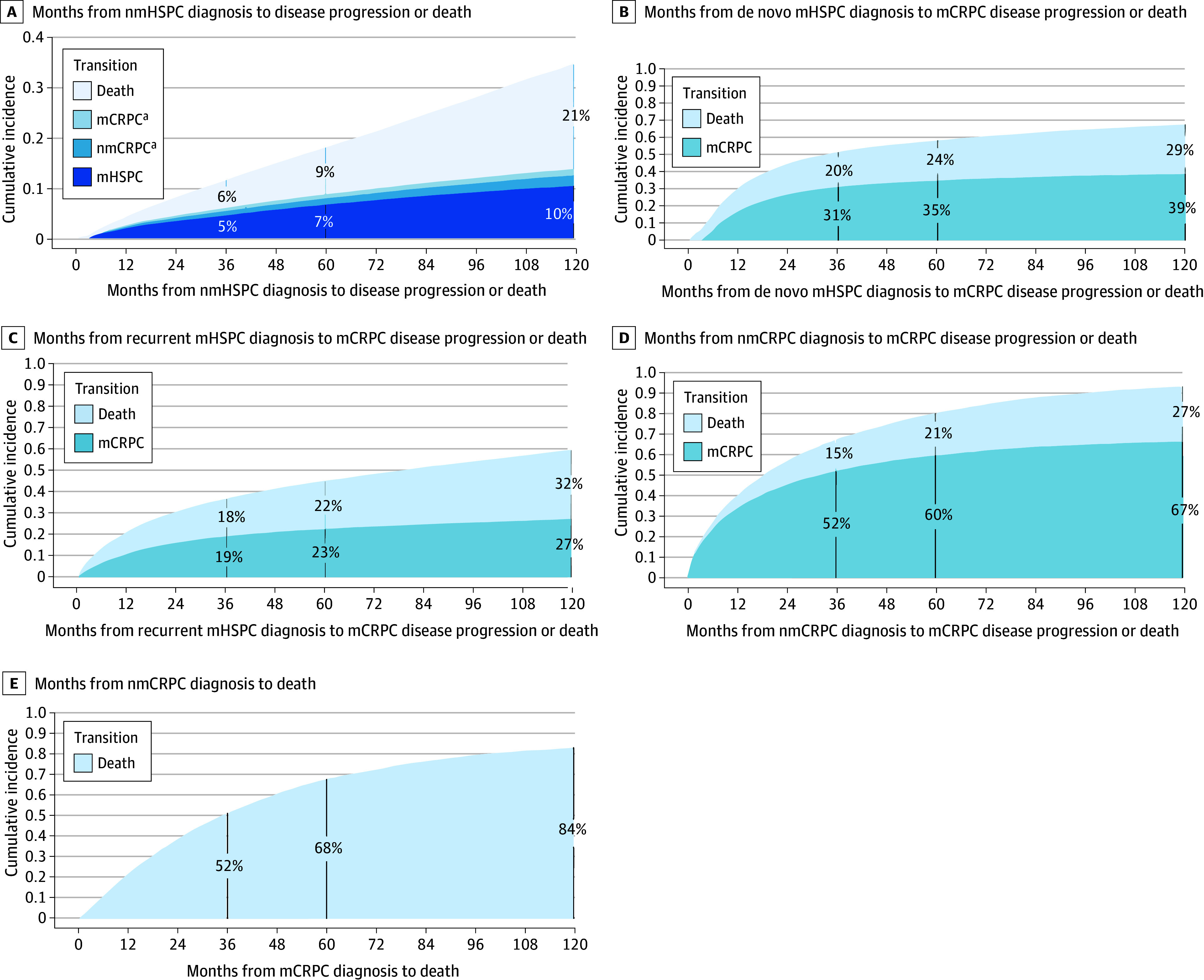
Cumulative Incidence Curves for the Time From Diagnosis of a Disease State to Disease Progression or Death as Competing Risks mCRPC indicates metastatic castrate-resistant prostate cancer; mHSPC, metastatic hormone–sensitive prostate cancer; nmCRPC, nonmetastatic castrate-resistant prostate cancer; nmHSPC, nonmetastatic hormone-sensitive prostate cancer. ^a^Cumulative incidence curve estimates for mCRPC and nmCRPC are less than 3% at all time points; note the y-axis ranges from 0 to 0.4.

Figure 3 presents cumulative incidence curves for transitions from less to more advanced PC disease states or death before observable disease progression, treating these events as competing risks. In nmHSPC, progression rates were low and minimally increased after 5 years. By 10 years, 10% of patients progressed to recurrent mHSPC, 2% progressed to nmCRPC, and 1% progressed directly to mCRPC without detection of an intermediate disease state; 21% of patients died (eTables 12-16 in Supplement 1). Adjusting for age, progression risk to recurrent mHSPC was significantly higher for Black (HR, 1.36; 95% CI, 1.33-1.40) and Hispanic (HR, 1.38; 95% CI, 1.31-1.45) vs White patients (Table 2). Similarly, progression risk to nmCRPC was significantly higher for Black (HR, 1.60; 95% CI, 1.51-1.70) and Hispanic (HR, 1.55; 95% CI, 1.40-1.72) vs White patients.

**Table 2.** HRs for the Time From Diagnosis of a Disease State to Disease Progression or Death as Competing Risks

Disease state, transition, and race	HR (95% CI)^a^	*P* value
nmHSPC		
mHSPC		
Black	1.36 (1.33-1.40)	<.001
Hispanic	1.38 (1.31-1.45)	<.001
White	1 [Reference]	NA
nmCRPC		
Black	1.60 (1.51-1.70)	<.001
Hispanic	1.55 (1.40-1.72)	<.001
White	1 [Reference]	NA
mCRPC		
Black	0.69 (0.63-0.76)	<.001
Hispanic	0.57 (0.47-0.69)	<.001
White	1 [Reference]	NA
Death		
Black	1.02 (1.00-1.04)	.03
Hispanic	0.81 (0.78-0.83)	<.001
White	1 [Reference]	NA
De novo mHSPC		
mCRPC		
Black	1.00 (0.94-1.06)	.99
Hispanic	0.90 (0.80-1.02)	.09
White	1 [Reference]	NA
Death		
Black	1.00 (0.93-1.07)	.96
Hispanic	1.01 (0.89-1.14)	.92
White	1 [Reference]	NA
Recurrent mHSPC		
mCRPC		
Black	1.03 (0.97-1.09)	.37
Hispanic	0.87 (0.77-0.97)	.01
White	1 [Reference]	NA
Death		
Black	0.91 (0.86-0.96)	.001
Hispanic	0.98 (0.89-1.08)	.69
White	1 [Reference]	NA
nmCRPC		
mCRPC		
Black	0.83 (0.78-0.89)	<.001
Hispanic	0.88 (0.79-0.99)	.04
White	1 [Reference]	NA
Death		
Black	0.99 (0.89-1.10)	.91
Hispanic	0.95 (0.79-1.14)	.56
White	1 [Reference]	NA
mCRPC, death		
Black	0.84 (0.81-0.88)	<.001
Hispanic	0.76 (0.69-0.83)	<.001
White	1 [Reference]	NA

Abbreviations: HR, hazard ratio; mCRPC, metastatic castrate-resistant prostate cancer; mHSPC, metastatic hormone-sensitive prostate cancer; NA, not applicable; nmCRPC, nonmetastatic castrate-resistant prostate cancer; nmHSPC, nonmetastatic hormone-sensitive prostate cancer.

^a^ The HRs were obtained from Fine and Gray competing risk models that adjusted for age at diagnosis.

For de novo mHSPC, 5-year estimates showed that 35% of patients progressed to mCRPC and 24% died. In recurrent mHSPC, 23% of patients progressed to mCRPC and 22% died by 5 years. Adjustments for age, race, and ethnicity were not significantly associated with mCRPC progression or death in de novo mHSPC. However, in recurrent mHSPC, Hispanic patients had a lower mCRPC progression risk (HR, 0.87; 95% CI, 0.77-0.97), whereas Black patients’ risk was similar to that of White patients (HR, 1.03; 95% CI, 0.97-1.09). Long-term follow-up revealed a subgroup of patients with mHSPC who had favorable long-term progression-free survival, with rates increasing modestly from 5-year to 10-year follow-up (39% for de novo and 27% for recurrent disease).

In nmCRPC, 5-year estimates showed that 60% of patients progressed to mCRPC and 21% died. After adjusting for age, Black (HR, 0.83; 95% CI, 0.78-0.89) and Hispanic (HR, 0.88; 95% CI, 0.79-0.99) patients had a lower mCRPC progression risk than White patients, but there was no significant association with death.

For mCRPC, the 5-year estimate for death from any cause was 68%. After adjusting for age, death risk was lower for Black (HR, 0.84; 95% CI, 0.81-0.88) and Hispanic (HR, 0.76; 95% CI, 0.69-0.83) patients vs White patients.

## Discussion

The clinical landscape of PC has changed dramatically over the past 15 years, and the impact on the epidemiology of PC across disease states and race and ethnicity has not been well-documented. This cohort study of 6 539 001 veterans is one of the largest longitudinal investigations of PC epidemiology to date, spanning 10 years from 2012 to 2021. We characterized incidence, prevalence, and disease progression rates across the disease continuum and examined racial and ethnic differences. Depending on year and disease state, there was a 2-fold to 4-fold higher incidence and a 2-fold to 5.5-fold higher prevalence of PC among Black vs White patients. Although race and ethnicity were associated with progression in nmHSPC, in nmCRPC and mCRPC, Black and Hispanic patients had lower progression risks.

The temporal trends for nmHSPC IRs, including the 2016 nadir, align with shifts in screening practices following the 2012 US Preventive Services Task Force guidelines advising against PSA screening and its 2017 draft reversal.^1,23^ The increase in IRs for metastatic PC, including both de novo and recurrent mHSPC, throughout the study is consistent with the findings of Desai et al,^11^ who used Surveillance, Epidemiology, and End Results data. These trends may be partially attributable to decreases in PSA testing, particularly in de novo disease, and uptake in positron emission tomography–based imaging, which are used most commonly in recurrent HSPC disease.^2^

Changes in nmCRPC and mCRPC, disease states that emerge after treatment, are less likely to be associated with PSA screening practices. Before 2011, chemotherapy was the only life-prolonging therapy for mCRPC. It is possible that increasing treatment options led to more-aggressive efforts to detect metastatic cancer after 2012, contributing to the increased incidence of mCRPC.^24^

Factors related to racial disparities may impact outcomes differently at each point along the PC continuum, calling for an examination of disparities at each stage.^25^ Our study uniquely provides IR estimates of disease states by CR status.^11,13^ Yamoah et al^12^ highlighted racial differences in PC incidence between Black and White veterans from 2005 to 2019, noting that Black patients had twice the incidence of both localized and de novo metastatic disease. Our findings support this within nmHSPC and de novo mHSPC and suggest that these differences extend to more-advanced states, including recurrent mHSPC, nmCRPC, and mCRPC. We also found that Hispanic ethnicity was associated with a 1.1-fold to 1.5-fold increased incidence vs White patients, with larger disparities in more-advanced states.

We provided detailed general population data on disease progression over long-term follow-up. For nmHSPC, 10-year disease progression rates of 13% and all-cause mortality rates of 21% align with estimates by Yamoah et al,^12^ who found a 10% 10-year cumulative incidence for metastatic disease using a treatment-stratified model adjusting for age, PSA level, Gleason score, time to treatment, ADT use, and diagnosis year.

In mHSPC, 5-year mCRPC progression rates of 35% for de novo disease and 23% for recurrent disease closely match those of previous trials.^26,27^ Notably, our long-term follow-up revealed a sizable subgroup with mHSPC showing favorable long-term progression-free survival, with rates increasing modestly from 5-year to 10-year follow-up (39% for de novo and 27% for recurrent disease). This challenges the perception of metastases as invariably terminal, although potential misdiagnoses of metastatic disease due to misclassification from imaging results may occur.

Our study highlights that Black race and Hispanic ethnicity are associated with greater progression risk from nmHSPC to mHSPC and nmCRPC. Of note, progression is not influenced solely by disease aggressiveness but also by treatments received. Yamoah et al^12^ found that Black patients receiving definitive primary treatment had lower metastases risks, but found increased risk among those undergoing active surveillance or without primary treatment. Given the increased risk associated with Hispanic patients in our study, and the fact that Hispanic patients are much less well studied than Black patients, future studies should explore how treatment may influence results for Hispanic patients.

We also found that Black race and Hispanic ethnicity were associated with a reduced progression risk from nmCRPC to mCRPC, and from mCRPC to death. This inverse association of Black race and Hispanic ethnicity aligns with results of Rasmussen et al,^25^ who analyzed racial and ethnic differences in time from nmCRPC diagnosis to mCRPC progression or death among veterans, and prior work from our team.^28^ Our findings support that Black and Hispanic patients may experience better outcomes vs White patients when receiving treatment in an equal-access health care setting, and indicate that this trend may extend to other advanced disease states.

### Strengths and Limitations

This is one of the largest and most comprehensive studies of PC epidemiology to date. The VHA provides an ideal setting for studying racial and ethnic disparities in health care owing to its diverse population and fully integrated medical records. The VHA’s equal-access system created a relatively controlled environment for assessing disparities that extend beyond access to care and medication costs.

Despite these strengths, this study has limitations. The findings from veterans may not generalize to the broader population. In addition, because this was a claims-based study relying on unstructured data for patient classification into disease states, some misclassifications may have occurred. However, misclassification should not be associated with race and would not explain our findings regarding racial disparities. We used validated algorithms to detect metastatic disease and censored patients who developed non-PC malignant tumors to reduce misclassification. Additionally, the lack of treatment data may have confounded observed racial and ethnic differences, and our follow-up period ended before the approval of newer therapies for mCRPC so we could not assess their impact. The exact causes of racial differences observed in this study are likely multifactorial, requiring further research.^29,30^ Furthermore, race and ethnicity are social constructs, and further work is needed to fully capture social determinants of health beyond race and ethnicity to better understand the factors underlying disparities.

## Conclusions

Our findings provide novel data on IRs, prevalence, and progression rates across PC disease states by race and ethnicity within the VHA. The temporal trends for IRs and prevalence align with changes in PSA testing guidelines and increasing use of advanced imaging. Notably, in an environment of relatively equal access to care, Black patients experienced a disproportionate burden of PC, with IRs and prevalence rates more than 2-fold higher vs White patients. Interestingly, Black race and Hispanic ethnicity were associated with an increased progression risk within some earlier disease states but lower risk in more advanced disease states. These findings highlight the importance of evaluating racial and ethnic differences across the disease continuum and provide valuable data to guide clinical trial diversity efforts and inform target populations and follow-up strategies for end-point analysis.

## Notes

Supplement 1. **eTable 1.** Characteristics of the nmHSPC cohort, stratified by race and ethnicity **eTable 2.** Characteristics of the de novo mHSPC cohort, stratified by race and ethnicity **eTable 3.** Characteristics of the recurrent mHSPC cohort, stratified by race and ethnicity **eTable 4.** Characteristics of the nmCRPC cohort, stratified by race and ethnicity **eTable 5.** Characteristics of the mCRPC cohort, stratified by race and ethnicity **eTable 6.** Age-adjusted incidence rates (per 100K person-years) and 95% confidence intervals by disease state and year **eTable 7.** Age-adjusted incidence rates (per 100K person-years) and 95% confidence intervals by race/ethnicity, disease state, and year **eTable 8.** Annual percentage change (APC) in incidence rates **eTable 9.** Annual percentage change (APC) in point prevalence rates **eTable 10.** Age-adjusted point prevalence (per 100K) and 95% confidence intervals by disease state and year **eTable 11.** Age-adjusted point prevalence (per 100K) and 95% confidence intervals by race/ethnicity, disease state, and year **eFigure.** Age-adjusted point prevalence (per 100K person-years) by race/ethnicity, disease state, and year **eTable 12.** Cumulative incidence estimates of time from nmHSPC diagnosis to disease progression or death as competing risks **eTable 13.** Cumulative incidence estimates of time from de novo mHSPC diagnosis to mCRPC disease progression or death as competing risks **eTable 14.** Cumulative incidence estimates of time from recurrent mHSPC diagnosis to mCRPC disease progression or death as competing risks **eTable 15.** Cumulative incidence estimates of time from nmCRPC diagnosis to mCRPC disease progression or death as competing risks **eTable 16.** Cumulative incidence estimates of time from mCRPC diagnosis to death

Supplement 2.
Data Sharing Statement
