# Supplementary material for: Racial and Ethnic Differences in Prostate Cancer Epidemiology Across Disease States in the VA
Source: JAMA Netw Open. 2024 Nov 15;7(11):e2445505. doi: 10.1001/jamanetworkopen.2024.45505 (PMC11568464; doi:10.1001/jamanetworkopen.2024.45505)
Supplement: Supplement 1. — eTable 1. Characteristics of the nmHSPC cohort, stratified by race and ethnicity eTable 2. Characteristics of the de novo mHSPC cohort, stratified by race and ethnicity eTable 3. Characteristics of the recurrent mHSPC cohort, stratified by race and ethnicity eTable 4. Characteristics of the nmCRPC cohort, stratified by race and ethnicity eTable 5. Characteristics of the mCRPC cohort, stratified by race and ethnicity eTable 6. Age-adjusted incidence rates (per 100K person-years) and 95% confidence intervals by disease state and year eTable 7. Age-adjusted incidence rates (per 100K person-years) and 95% confidence intervals by race/ethnicity, disease state, and year eTable 8. Annual percentage change (APC) in incidence rates eTable 9. Annual percentage change (APC) in point prevalence rates eTable 10. Age-adjusted point prevalence (per 100K) and 95% confidence intervals by disease state and year eTable 11. Age-adjusted point prevalence (per 100K) and 95% confidence intervals by race/ethnicity, disease state, and year eFigure. Age-adjusted point prevalence (per 100K person-years) by race/ethnicity, disease state, and year eTable 12. Cumulative incidence estimates of time from nmHSPC diagnosis to disease progression or death as competing risks eTable 13. Cumulative incidence estimates of time from de novo mHSPC diagnosis to mCRPC disease progression or death as competing risks eTable 14. Cumulative incidence estimates of time from recurrent mHSPC diagnosis to mCRPC disease progression or death as competing risks eTable 15. Cumulative incidence estimates of time from nmCRPC diagnosis to mCRPC disease progression or death as competing risks eTable 16. Cumulative incidence estimates of time from mCRPC diagnosis to death [file jamanetwopen-e2445505-s001.pdf]

Supplemental Online Content

Stock SR, Burns MT, Waller J, et al. Racial and ethnic differences in prostate cancer epidemiology across disease states in the VA. *JAMA Netw Open*. 2024;7(11):e2445505. doi:10.1001/jamanetworkopen.2024.45505

eTable 1. Characteristics of the nmHSPC cohort, stratified by race and ethnicity

eTable 2. Characteristics of the de novo mHSPC cohort, stratified by race and ethnicity

eTable 3. Characteristics of the recurrent mHSPC cohort, stratified by race and ethnicity

eTable 4. Characteristics of the nmCRPC cohort, stratified by race and ethnicity

eTable 5. Characteristics of the mCRPC cohort, stratified by race and ethnicity

eTable 6. Age-adjusted incidence rates (per 100K person-years) and 95% confidence intervals by disease state and year

eTable 7. Age-adjusted incidence rates (per 100K person-years) and 95% confidence intervals by race/ethnicity, disease state, and year

eTable 8. Annual percentage change (APC) in incidence rates

eTable 9. Annual percentage change (APC) in point prevalence rates

eTable 10. Age-adjusted point prevalence (per 100K) and 95% confidence intervals by disease state and year

eTable 11. Age-adjusted point prevalence (per 100K) and 95% confidence intervals by race/ethnicity, disease state, and year

eFigure. Age-adjusted point prevalence (per 100K person-years) by race/ethnicity, disease state, and year

eTable 12. Cumulative incidence estimates of time from nmHSPC diagnosis to disease progression or death as competing risks

eTable 13. Cumulative incidence estimates of time from de novo mHSPC diagnosis to mCRPC disease progression or death as competing risks

eTable 14. Cumulative incidence estimates of time from recurrent mHSPC diagnosis to mCRPC disease progression or death as competing risks

eTable 15. Cumulative incidence estimates of time from nmCRPC diagnosis to mCRPC disease progression or death as competing risks

eTable 16. Cumulative incidence estimates of time from mCRPC diagnosis to death

This supplemental material has been provided by the authors to give readers additional information about their work.

© 2024 Stock SR et al. *JAMA Network Open.*

eTable 1. Characteristics of the nmHSPC cohort*, stratified by race and ethnicity.

	Black (N=88888)	Hispanic (N=20001)	White (N=324482)	p-value (Black vs. White)	p-value (Hispanic vs. White)
**Age at Diagnosis of disease state**				<0.001	<0.001
Median	64.6	68.4	70.0		
Q1, Q3	59.3, 70.2	62.8, 74.5	64.5, 76.0		
**PSA at Diagnosis of disease state**				<0.001	<0.001
Missing	38651	9385	161616		
Median	5.7	5.2	4.5		
Q1, Q3	2.7, 9.4	1.5, 8.4	0.3, 7.4		
**Total Gleason Score at PC diagnosis**				<0.001	<0.001
< 6	45 (0.2%)	12 (0.3%)	202 (0.4%)		
6	8641 (36.5%)	1634 (41.2%)	19253 (39.1%)		
7 (3 + 4)	7994 (33.7%)	1143 (28.8%)	14737 (29.9%)		
7 (4 + 3)	3290 (13.9%)	532 (13.4%)	6341 (12.9%)		
8	2061 (8.7%)	380 (9.6%)	4495 (9.1%)		
9-10	1671 (7.1%)	268 (6.8%)	4230 (8.6%)		
Missing	65186	16032	275224		
**Site of Metastasis at Metastatic Diagnosis**					
Missing	88888	20001	324482		

*Note: Includes patients who were part of the nmHSPC cohort at any point during the study period
** Other includes Alaskan Native, Asian, Native American, and Pacific Islander patients of non-Hispanic ethnicity
Abbreviations: PC = Prostate Cancer, nmHSPC = non-metastatic hormone-sensitive prostate cancer, PSA= Prostate Specific Antigen, CCI= Charlson Comorbidity Index

© 2024 Stock SR et al. *JAMA Network Open.*

eTable 2. Characteristics of the de novo mHSPC cohort*, stratified by race and ethnicity.

	Black (N=4929)	Hispanic (N=1025)	White (N=11684)	p-value (Black vs. White)	p-value (Hispanic vs. White)
**Age at Diagnosis of disease state**				<0.001	0.34
Median	66.7	71.2	71.5		
Q1, Q3	61.3, 74.0	64.6, 80.2	65.7, 79.6		
**PSA at Diagnosis of disease state**				<0.001	0.22
Missing	1612	370	4078		
Median	26.5	19.9	18.6		
Q1, Q3	8.0, 156.2	6.8, 115.5	6.6, 98.0		
**Total Gleason Score at PC diagnosis**				<0.001	<0.001
< 6	2 (0.1%)	0 (0.0%)	5 (0.1%)		
6	187 (11.1%)	55 (16.7%)	325 (9.5%)		
7 (3 + 4)	339 (20.1%)	76 (23.0%)	529 (15.5%)		
7 (4 + 3)	245 (14.5%)	28 (8.5%)	423 (12.4%)		
8	353 (20.9%)	68 (20.6%)	709 (20.8%)		
9-10	564 (33.4%)	103 (31.2%)	1416 (41.6%)		
Missing	3239	695	8277		
**Site of Metastasis at Metastatic Diagnosis**				0.003	0.73
Bone	155 (60.5%)	29 (56.9%)	500 (55.8%)		
Liver	5 (2.0%)	0 (0.0%)	9 (1.0%)		
Lung	8 (3.1%)	1 (2.0%)	22 (2.5%)		
Lymph	46 (18.0%)	9 (17.6%)	119 (13.3%)		
Multiple Sites	13 (5.1%)	2 (3.9%)	38 (4.2%)		
Other/Unspecified	29 (11.3%)	10 (19.6%)	208 (23.2%)		
Missing	4673	974	10788		

*Note: Includes patients who were part of the de novo mHSPC cohort at any point during the study period.
** Other includes Alaskan Native, Asian, Native American, and Pacific Islander patients of non-Hispanic ethnicity
Abbreviations: PC = Prostate Cancer, mHSPC = metastatic hormone-sensitive prostate cancer, PSA= Prostate Specific Antigen, CCI= Charlson Comorbidity Index

© 2024 Stock SR et al. *JAMA Network Open.*

eTable 3. Characteristics of the recurrent mHSPC cohort*, stratified by race and ethnicity.

	Black (N=9623)	Hispanic (N=2049)	White (N=24004)	p-value (Black vs. White)	p-value (Hispanic vs. White)
**Age at Diagnosis of disease state**				<0.001	0.004
Median	69.6	74.4	73.4		
Q1, Q3	64.2, 75.7	68.2, 82.3	68.3, 80.5		
**PSA at Diagnosis of disease state**				<0.001	0.28
Missing	3496	836	9871		
Median	3.5	2.3	2.6		
Q1, Q3	0.3, 15.7	0.2, 11.1	0.2, 12.7		
**Total Gleason Score at PC diagnosis**				<0.001	<0.001
< 6	8 (0.3%)	3 (0.6%)	26 (0.4%)		
6	931 (30.1%)	194 (36.1%)	1802 (30.2%)		
7 (3 + 4)	905 (29.2%)	137 (25.5%)	1562 (26.2%)		
7 (4 + 3)	472 (15.2%)	82 (15.3%)	853 (14.3%)		
8	376 (12.1%)	74 (13.8%)	776 (13.0%)		
9-10	405 (13.1%)	47 (8.8%)	950 (15.9%)		
Missing	6526	1512	18035		
**Site of Metastasis at Metastatic Diagnosis**				0.003	0.13
Bone	145 (50.2%)	31 (47.7%)	573 (51.9%)		
Liver	16 (5.5%)	1 (1.5%)	27 (2.4%)		
Lung	15 (5.2%)	2 (3.1%)	49 (4.4%)		
Lymph	74 (25.6%)	10 (15.4%)	222 (20.1%)		
Multiple Sites	7 (2.4%)	5 (7.7%)	27 (2.4%)		
Other/Unspecified	32 (11.1%)	16 (24.6%)	207 (18.7%)		
Missing	9334	1984	22899		

*Note: Includes patients who were part of the recurrent mHSPC cohort at any point during the study period.
**Other includes Alaskan Native, Asian, Native American, and Pacific Islander patients of non-Hispanic ethnicity
Abbreviations: PC = Prostate Cancer, mHSPC = metastatic hormone-sensitive prostate cancer, PSA= Prostate Specific Antigen, CCI= Charlson Comorbidity Index

© 2024 Stock SR et al. *JAMA Network Open.*

eTable 4. Characteristics of the nmCRPC cohort*, stratified by race and ethnicity.

	Black (N=2317)	Hispanic (N=531)	White (N=5364)	p-value (Black vs. White)	p-value (Hispanic vs. White)
**Age at Diagnosis of disease state**				<0.001	0.78
Median	73.7	78.7	77.8		
Q1, Q3	66.7, 81.7	71.2, 84.7	70.9, 85.2		
**PSA at Diagnosis of disease state**				0.36	<0.001
Missing	151	18	508		
Median	5.7	4.6	5.5		
Q1, Q3	3.3, 11.7	3.0, 9.2	3.3, 11.8		
**Total Gleason Score at PC diagnosis**				<0.001	0.47
< 6	0 (0.0%)	0 (0.0%)	4 (0.4%)		
6	76 (12.9%)	17 (16.0%)	119 (10.6%)		
7 (3 + 4)	134 (22.8%)	17 (16.0%)	176 (15.7%)		
7 (4 + 3)	94 (16.0%)	18 (17.0%)	161 (14.4%)		
8	93 (15.8%)	21 (19.8%)	242 (21.6%)		
9-10	192 (32.6%)	33 (31.1%)	419 (37.4%)		
Missing	1728	425	4243		
**Site of Metastasis at Metastatic Diagnosis**					
Missing	2317	531	5364		

*Note: Includes patients who were part of the nmCRPC cohort at any point during the study period.
** Other includes Alaskan Native, Asian, Native American, and Pacific Islander patients of non-Hispanic ethnicity
Abbreviations: PC = Prostate Cancer, nmCRPC = non-metastatic castrate-resistant prostate cancer, PSA= Prostate Specific Antigen, CCI= Charlson Comorbidity Index

© 2024 Stock SR et al. *JAMA Network Open.*

eTable 5. Characteristics of the mCRPC cohort*, stratified by race and ethnicity.

	Black (N=5598)	Hispanic (N=1144)	White (N=15538)	p-value (Black vs. White)	p-value (Hispanic vs. White)
**Age at Diagnosis of disease state**				<0.001	0.81
Median	71.1	76.1	75.6		
Q1, Q3	65.2, 79.4	69.1, 83.6	69.5, 83.3		
**PSA at Diagnosis of disease state**				<0.001	0.63
Missing	588	159	3070		
Median	10.8	9.2	9.1		
Q1, Q3	3.3, 48.2	3.3, 32.7	3.0, 35.1		0.006
**Total Gleason Score at PC diagnosis**				<0.001	
< 6	2 (0.1%)	1 (0.3%)	10 (0.3%)		
6	140 (8.3%)	33 (11.3%)	262 (6.8%)		
7 (3 + 4)	238 (14.1%)	40 (13.7%)	457 (11.8%)		
7 (4 + 3)	239 (14.2%)	42 (14.4%)	468 (12.1%)		
8	319 (19.0%)	67 (23.0%)	849 (21.9%)		
9-10	745 (44.3%)	108 (37.1%)	1833 (47.3%)		
Missing	3915	853	11659		
**Site of Metastasis at Metastatic Diagnosis**				0.06	0.10
Bone	171 (65.8%)	37 (75.5%)	588 (72.1%)		
Liver	1 (0.4%)	0 (0.0%)	4 (0.5%)		
Lung	5 (1.9%)	0 (0.0%)	12 (1.5%)		
Lymph	54 (20.8%)	11 (22.4%)	105 (12.9%)		
Multiple Sites	12 (4.6%)	1 (2.0%)	37 (4.5%)		
Other/Unspecified	17 (6.5%)	0 (0.0%)	69 (8.5%)		
Missing	5338	1095	14723		

*Note: Includes patients who were part of the mCRPC cohort at any point during the study period.
** Other includes Alaskan Native, Asian, Native American, and Pacific Islander patients of non-Hispanic ethnicity
Abbreviations: PC = Prostate Cancer, mCRPC = metastatic castrate-resistant prostate cancer, PSA= Prostate Specific Antigen, CCI= Charlson Comorbidity Index

© 2024 Stock SR et al. *JAMA Network Open.*

eTable 6. Age-adjusted incidence rates (per 100K person-years) and 95% confidence intervals by disease state and year.

**Disease state**	**2012**	**2013**	**2014**	**2015**	**2016**	**2017**	**2018**	**2019**	**2020**
**nmHSPC**	389.68 (382.11, 397.25)	344.30 (337.45, 351.15)	323.85 (317.34, 330.37)	292.01 (285.68, 298.34)	261.49 (255.49, 267.48)	266.13 (260.20, 272.06)	308.50 (302.23, 314.77)	287.19 (281.29, 293.08)	218.89 (213.81, 223.96)
**Overall mHSPC**	39.98 (37.84, 42.12)	39.44 (37.38, 41.50)	44.51 (42.33, 46.70)	46.28 (44.15, 48.42)	47.24 (45.17, 49.30)	50.16 (48.03, 52.30)	57.72 (55.53, 59.92)	59.09 (56.95, 61.23)	50.05 (48.15, 51.96)
**De novo**	17.42 (15.91, 18.93)	16.33 (14.95, 17.71)	18.66 (17.08, 20.24)	18.22 (16.72, 19.72)	19.36 (17.87, 20.86)	20.57 (19.03, 22.10)	23.62 (22.12, 25.12)	24.91 (23.37, 26.45)	19.40 (18.10, 20.71)
**Recurrent**	23.55 (22.00, 25.10)	24.07 (22.52, 25.62)	26.87 (25.33, 28.41)	29.09 (27.54, 30.63)	28.99 (27.54, 30.45)	30.75 (29.23, 32.26)	35.55 (33.92, 37.19)	35.71 (34.18, 37.23)	31.88 (30.46, 33.30)
**nmCRPC**	6.99 (6.24, 7.75)	6.04 (5.42, 6.65)	5.48 (4.86, 6.10)	4.97 (4.48, 5.46)	5.61 (5.03, 6.18)	6.88 (6.15, 7.60)	7.06 (6.47, 7.65)	9.27 (8.54, 10.00)	4.71 (4.21, 5.21)
**mCRPC**	15.94 (14.71, 17.17)	17.92 (16.78, 19.07)	17.47 (16.53, 18.42)	19.59 (18.35, 20.82)	21.12 (19.87, 22.38)	27.08 (25.69, 28.47)	20.01 (18.90, 21.12)	21.78 (20.59, 22.97)	16.12 (15.11, 17.14)

© 2024 Stock SR et al. *JAMA Network Open.*

eTable 7. Age-adjusted incidence rates (per 100K person-years) and 95% confidence intervals by race/ethnicity, disease state, and year.

Disease state	2012	2013	2014	2015	2016	2017	2018	2019	2020
**nmHSPC**									
Non-Hispanic White	320.84 (313.01, 328.67)	275.08 (268.27, 281.89)	263.03 (256.21, 269.86)	226.22 (219.86, 232.57)	199.88 (193.70, 206.06)	207.05 (200.89, 213.21)	244.19 (237.60, 250.78)	225.40 (219.23, 231.57)	169.99 (164.80, 175.18)
Non-Hispanic Black	671.53 (649.85, 693.21)	621.90 (601.74, 642.05)	581.20 (562.28, 600.12)	541.30 (522.51, 560.09)	501.34 (483.40, 519.29)	500.41 (482.44, 518.38)	555.58 (536.89, 574.28)	536.02 (517.82, 554.22)	391.38 (375.82, 406.94)
RR (vs. White)	2.09 (2.01, 2.18) P < 0.001	2.26 (2.17, 2.35) P < 0.001	2.21 (2.12, 2.30) P < 0.001	2.39 (2.29, 2.50) P < 0.001	2.51 (2.39, 2.63) P < 0.001	2.42 (2.31, 2.53) P < 0.001	2.28 (2.18, 2.38) P < 0.001	2.38 (2.28, 2.48) P < 0.001	2.30 (2.19, 2.42) P < 0.001
Hispanic	325.88 (300.41, 351.35)	295.24 (270.91, 319.56)	286.89 (264.18, 309.61)	267.87 (246.49, 289.25)	222.05 (203.37, 240.73)	214.78 (198.09, 231.47)	250.84 (232.43, 269.25)	232.43 (215.76, 249.11)	202.30 (186.00, 218.61)
RR (vs. White)	1.02 (0.94, 1.10) P = 0.71	1.07 (0.98, 1.17) P = 0.11	1.09 (1.00, 1.19) P = 0.04	1.18 (1.09, 1.29) P < 0.001	1.11 (1.02, 1.22) P = 0.02	1.04 (0.95, 1.13) P = 0.39	1.03 (0.95, 1.11) P = 0.50	1.03 (0.95, 1.11) P = 0.43	1.19 (1.09, 1.30) P < 0.001
**Overall mHSPC**									
Non-Hispanic White	32.29 (29.90, 34.68)	30.25 (28.22, 32.28)	33.19 (31.10, 35.29)	35.13 (32.97, 37.29)	36.09 (33.96, 38.21)	37.96 (35.82, 40.11)	45.15 (42.83, 47.47)	46.54 (44.28, 48.80)	39.05 (37.11, 40.99)
Non-Hispanic Black	83.26 (76.85, 89.68)	84.91 (78.28, 91.54)	95.10 (88.03, 102.18)	97.89 (91.04, 104.73)	104.72 (97.77, 111.66)	104.40 (97.46, 111.33)	118.14 (111.03, 125.24)	118.16 (111.23, 125.10)	98.70 (92.39, 105.00)
RR (vs. White)	2.58 (2.32, 2.87) P < 0.001	2.81 (2.53, 3.11) P < 0.001	2.87 (2.60, 3.16) P < 0.001	2.79 (2.54, 3.06) P < 0.001	2.90 (2.66, 3.17) P < 0.001	2.75 (2.52, 3.00) P < 0.001	2.62 (2.42, 2.83) P < 0.001	2.54 (2.35, 2.74) P < 0.001	2.53 (2.33, 2.74) P < 0.001
Hispanic	44.97 (36.75, 53.18)	38.17 (31.78, 44.55)	43.21 (36.29, 50.12)	49.35 (41.95, 56.74)	45.83 (39.04, 52.62)	56.84 (49.17, 64.51)	58.93 (51.15, 66.70)	59.69 (52.23, 67.16)	49.82 (43.46, 56.19)
RR (vs. White)	1.39 (1.14, 1.70) P < 0.001	1.26 (1.05, 1.51) P = 0.01	1.30 (1.10, 1.55) P = 0.003	1.40 (1.19, 1.65) P < 0.001	1.27 (1.08, 1.49) P = 0.003	1.50 (1.29, 1.73) P < 0.001	1.31 (1.13, 1.50) P < 0.001	1.28 (1.12, 1.47) P < 0.001	1.28 (1.11, 1.46) P < 0.001

© 2024 Stock SR et al. *JAMA Network Open.*

Disease state	2012	2013	2014	2015	2016	2017	2018	2019	2020
**De novo mHSPC**									
Non-Hispanic White	13.43 (11.79, 15.07)	12.37 (11.04, 13.70)	14.18 (12.57, 15.79)	13.56 (12.01, 15.11)	14.03 (12.54, 15.52)	15.88 (14.28, 17.49)	19.13 (17.50, 20.76)	20.20 (18.50, 21.91)	14.82 (13.52, 16.12)
Non-Hispanic Black	40.37 (35.63, 45.11)	37.54 (32.88, 42.19)	37.77 (32.89, 42.65)	41.95 (37.04, 46.86)	44.44 (39.38, 49.51)	41.50 (36.70, 46.30)	45.69 (40.95, 50.43)	48.19 (43.47, 52.92)	39.45 (35.01, 43.89)
RR (vs. White)	3.01 (2.54, 3.56) P < 0.001	3.03 (2.58, 3.58) P < 0.001	2.66 (2.24, 3.16) P < 0.001	3.09 (2.63, 3.64) P < 0.001	3.17 (2.71, 3.70) P < 0.001	2.61 (2.24, 3.05) P < 0.001	2.39 (2.09, 2.73) P < 0.001	2.39 (2.10, 2.71) P < 0.001	2.66 (2.31, 3.07) P < 0.001
Hispanic	19.39 (13.95, 24.83)	14.96 (10.68, 19.23)	22.29 (16.45, 28.13)	16.73 (12.44, 21.01)	18.13 (13.72, 22.54)	21.73 (16.83, 26.62)	20.79 (15.71, 25.88)	27.37 (21.53, 33.22)	18.50 (14.73, 22.26)
RR (vs. White)	1.44 (1.06, 1.96) P = 0.02	1.21 (0.89, 1.64) P = 0.22	1.57 (1.18, 2.09) P = 0.002	1.23 (0.93, 1.63) P = 0.14	1.29 (0.99, 1.68) P = 0.06	1.37 (1.07, 1.75) P = 0.01	1.09 (0.84, 1.41) P = 0.53	1.35 (1.08, 1.70) P = 0.01	1.25 (1.00, 1.56) P = 0.05
**Recurrent mHSPC**									
Non-Hispanic White	19.58 (17.81, 21.34)	18.59 (17.04, 20.15)	19.77 (18.40, 21.14)	22.30 (20.77, 23.83)	22.84 (21.30, 24.38)	22.92 (21.47, 24.37)	27.12 (25.44, 28.80)	27.47 (25.95, 28.99)	25.15 (23.68, 26.62)
Non-Hispanic Black	47.21 (42.49, 51.93)	51.31 (46.27, 56.35)	60.91 (55.52, 66.30)	60.34 (55.23, 65.45)	64.86 (59.76, 69.96)	67.26 (61.95, 72.57)	77.44 (71.85, 83.04)	75.51 (70.09, 80.93)	63.50 (58.75, 68.26)
RR (vs. White)	2.41 (2.11, 2.76) P < 0.001	2.76 (2.43, 3.14) P < 0.001	3.08 (2.75, 3.45) P < 0.001	2.71 (2.43, 3.02) P < 0.001	2.84 (2.56, 3.15) P < 0.001	2.93 (2.65, 3.25) P < 0.001	2.86 (2.60, 3.14) P < 0.001	2.75 (2.51, 3.01) P < 0.001	2.52 (2.30, 2.78) P < 0.001
Hispanic	26.93 (20.60, 33.26)	24.27 (19.36, 29.17)	22.33 (18.34, 26.31)	33.87 (27.69, 40.05)	28.89 (23.57, 34.22)	36.68 (30.60, 42.77)	39.42 (33.39, 45.46)	34.05 (29.15, 38.95)	32.63 (27.34, 37.92)
RR (vs. White)	1.38 (1.07, 1.77) P = 0.01	1.31 (1.05, 1.62) P = 0.02	1.13 (0.93, 1.37) P = 0.21	1.52 (1.25, 1.85) P < 0.001	1.26 (1.04, 1.54) P = 0.02	1.60 (1.34, 1.91) P < 0.001	1.45 (1.23, 1.71) P < 0.001	1.24 (1.06, 1.45) P = 0.006	1.30 (1.09, 1.54) P = 0.003

© 2024 Stock SR et al. *JAMA Network Open.*

Disease state	2012	2013	2014	2015	2016	2017	2018	2019	2020
**nmCRPC**									
Non-Hispanic White	5.47 (4.66, 6.28)	4.54 (3.99, 5.09)	4.03 (3.42, 4.63)	3.84 (3.40, 4.27)	4.38 (3.70, 5.06)	4.58 (4.02, 5.14)	5.40 (4.82, 5.98)	7.58 (6.88, 8.28)	3.41 (3.00, 3.81)
Non-Hispanic Black	17.12 (14.30, 19.94)	15.63 (13.09, 18.17)	14.77 (12.22, 17.31)	11.50 (9.48, 13.53)	13.08 (10.96, 15.21)	18.87 (15.95, 21.78)	16.99 (14.56, 19.43)	19.37 (16.55, 22.18)	11.23 (9.17, 13.30)
RR (vs. White)	3.13 (2.51, 3.90) P < 0.001	3.44 (2.81, 4.22) P < 0.001	3.67 (2.92, 4.61) P < 0.001	3.00 (2.43, 3.70) P < 0.001	2.98 (2.38, 3.74) P < 0.001	4.12 (3.39, 5.02) P < 0.001	3.15 (2.63, 3.77) P < 0.001	2.56 (2.15, 3.04) P < 0.001	3.30 (2.65, 4.10) P < 0.001
Hispanic	10.04 (7.37, 12.71)	9.75 (6.18, 13.32)	7.44 (5.15, 9.72)	8.04 (4.86, 11.22)	7.17 (4.91, 9.43)	8.02 (5.04, 10.99)	5.88 (3.92, 7.83)	6.12 (4.14, 8.09)	4.80 (3.02, 6.57)
RR (vs. White)	1.83 (1.35, 2.49) P < 0.001	2.15 (1.46, 3.16) P < 0.001	1.85 (1.31, 2.60) P < 0.001	2.09 (1.39, 3.16) P < 0.001	1.64 (1.15, 2.32) P = 0.006	1.75 (1.19, 2.59) P = 0.005	1.09 (0.77, 1.54) P = 0.63	0.81 (0.58, 1.13) P = 0.21	1.41 (0.95, 2.08) P = 0.09
**mCRPC**									
Non-Hispanic White	13.44 (12.04, 14.84)	14.83 (13.64, 16.03)	15.12 (14.13, 16.11)	16.17 (14.98, 17.35)	17.50 (16.11, 18.89)	22.65 (21.15, 24.16)	15.82 (14.61, 17.02)	17.22 (15.91, 18.54)	12.67 (11.69, 13.64)
Non-Hispanic Black	33.31 (29.29, 37.33)	36.89 (32.98, 40.80)	35.17 (31.59, 38.74)	39.63 (35.29, 43.98)	42.52 (38.40, 46.64)	55.13 (50.35, 59.92)	41.33 (37.52, 45.15)	44.49 (40.58, 48.39)	34.11 (30.39, 37.82)
RR (vs. White)	2.48 (2.11, 2.91) P < 0.001	2.49 (2.18, 2.84) P < 0.001	2.33 (2.06, 2.62) P < 0.001	2.45 (2.15, 2.80) P < 0.001	2.43 (2.14, 2.75) P < 0.001	2.43 (2.18, 2.71) P < 0.001	2.61 (2.32, 2.95) P < 0.001	2.58 (2.30, 2.90) P < 0.001	2.69 (2.36, 3.08) P < 0.001
Hispanic	14.73 (11.43, 18.03)	19.99 (15.48, 24.50)	13.46 (10.35, 16.57)	16.65 (12.59, 20.71)	22.78 (18.22, 27.34)	22.63 (18.65, 26.61)	19.07 (15.45, 22.69)	21.08 (16.70, 25.45)	14.07 (11.07, 17.08)
RR (vs. White)	1.10 (0.86, 1.40) P = 0.47	1.35 (1.06, 1.71) P = 0.01	0.89 (0.70, 1.13) P = 0.34	1.03 (0.80, 1.33) P = 0.82	1.30 (1.05, 1.62) P = 0.02	1.00 (0.83, 1.21) P = 0.99	1.21 (0.98, 1.48) P = 0.07	1.22 (0.98, 1.53) P = 0.07	1.11 (0.89, 1.39) P = 0.36

© 2024 Stock SR et al. *JAMA Network Open.*

eTable 8. Annual percentage change (APC) in incidence rates

Disease state	Race	Segment	APC	(95%CI)	p-value
nmHSPC	Overall	2012-2016	-9.1	(-15.7, -1.9)	0.03
		2016-2019	4.2	(-8.1, 18.1)	0.38
	Non-Hispanic Black	2012-2016	-7	(-11.2, -2.6)	0.02
		2016-2019	3	(-4.6, 11.2)	0.31
	Hispanic	2012-2017	-7.6	(-11.9, -3)	0.01
		2017-2019	4.7	(-13.7, 27.1)	0.50
	Non-Hispanic White	2012-2016	-10.5	(-18.1, -2.3)	0.03
		2016-2019	5.1	(-9.7, 22.4)	0.375
combined mHSPC	Overall	2012-2019	6.3	(4.9, 7.8)	<0.001
	Non-Hispanic Black	2012-2019	5.4	(4.2, 6.6)	<0.001
	Hispanic	2012-2019	6.3	(3.2, 9.5)	0.002
	Non-Hispanic White	2012-2016	3.9	(-4.8, 13.3)	0.26
		2016-2019	9.7	(-1.4, 22.1)	0.07
de novo	Overall	2012-2016	3.3	(-4.3, 11.5)	0.27
		2016-2019	9.6	(-0.6, 20.8)	0.06
	Non-Hispanic Black	2012-2019	3.1	(1.3, 5)	0.006
	Hispanic	2012-2019	5.5	(-0.5, 11.7)	0.07
	Non-Hispanic White	2012-2016	2.3	(-7.3, 12.9)	0.51
		2016-2019	13.7	(0.5, 28.6)	0.05
recurrent	overall	2012-2019	6.5	(5, 7.9)	<0.001
	Non-Hispanic Black	2012-2019	6.9	(4.9, 8.8)	<0.001
	Hispanic	2012-2019	6.8	(1.2, 12.7)	0.02
	Non-Hispanic White	2012-2019	6	(4.1, 8)	<0.001
nmCRPC	Overall	2012-2015	-11	(-24.8, 5.5)	0.12
		2015-2019	16.2	(6.4, 26.9)	0.01
	Non-Hispanic Black	2012-2015	-10.7	(-32.4, 18)	0.29
		2015-2019	12.6	(-4.1, 32.1)	0.1
	Hispanic	2012-2019	-6.8	(-9.9, -3.7)	0.002
	Non-Hispanic White	2012-2015	-12.1	(-31.2, 12.2)	0.19
		2015-2019	18.2	(4.2, 34.1)	0.02
mCRPC	overall	2012-2017	9.6	(5, 14.5)	0.007
		2017-2019	-8	(-21.7, 8.1)	0.20
	Non-Hispanic Black	2012-2017	9.1	(4.1, 14.3)	0.01
		2017-2019	-7.1	(-22, 10.6)	0.27
	Hispanic	2012-2019	5	(-1.4, 11.8)	0.11
	Non-Hispanic White	2012-2017	9.4	(6.9, 11.9)	0.001
		2017-2019	-10.9	(-18.4, -2.6)	0.03

© 2024 Stock SR et al. *JAMA Network Open.*

eTable 9. Annual percentage change (APC) in point prevalence rates

Disease state	Race	Segment	APC	(95%CI)	p-value
nmHSPC	Overall	2012-2017	-1.1	(-1.4, -0.9)	0.001
		2017-2019	1.2	(0.2, 2.2)	0.04
	Non-Hispanic Black	2012-2017	0.2	(-0.1, 0.5)	0.08
		2017-2019	1.5	(0.4, 2.7)	0.03
	Hispanic	2012-2017	-1.3	(-1.4, -1.1)	<0.001
		2017-2019	1.1	(0.5, 1.6)	0.008
	Non-Hispanic White	2012-2017	-1.8	(-2.1, -1.6)	<0.001
		2017-2019	1	(-0.1, 2)	0.06
Combined	Overall	2012-2017	8.1	(7.1, 9)	<0.001
		2017-2019	11.8	(8.5, 15.2)	0.001
	Non-Hispanic Black	2012-2019	8.2	(7.8, 8.6)	<0.001
	Hispanic	2012-2014	5	(-8.9, 21)	0.36
		2014-2019	10.5	(8, 13)	0.001
	Non-Hispanic White	2012-2014	4.7	(3.4, 6.3)	<0.001
		2014-2017	8	(7.2, 8.8)	<0.001
		2017-2019	13.6	(12.5, 14.7)	<0.001
de novo	Overall	2012-2017	5.6	(4.1, 7.1)	0.001
		2017-2019	10.4	(5.1, 16.1)	0.008
	Non-Hispanic Black	2012-2019	5.1	(4.4, 5.7)	<0.001
	Hispanic	2012-2016	4.7	(-2.8, 12.9)	0.15
		2016-2019	10.4	(1, 20.8)	0.04
	Non-Hispanic White	2012-2017	4.3	(3.3, 5.3)	0.001
		2017-2019	14.1	(10.3, 18)	0.001
Recurrent	Overall	2012-2016	9.2	(7.1, 11.4)	0.001
		2016-2019	11.9	(9.4, 14.4)	0.001
	Non-Hispanic Black	2012-2019	10.1	(9.6, 10.7)	<0.001
	Hispanic	2012-2019	10.7	(9.3, 12.2)	<0.001
	Non-Hispanic White	2012-2014	5.3	(4, 6.6)	<0.001
		2014-2017	10.1	(9.4, 10.8)	<0.001
		2017-2019	13.4	(12.5, 14.3)	<0.001
nmCRPC	Overall	2012-2016	-6	(-8.6, -3.4)	0.006
		2016-2019	16.1	(11.2, 21.3)	0.002
	Non-Hispanic Black	2012-2016	-7.4	(-9.6, -5.2)	0.002
		2016-2019	9.2	(5, 13.5)	0.005
	Hispanic	2012-2019	-3.7	(-4.4, -3)	<0.001
	Non-Hispanic White	2012-2014	-10.5	(-14.4, -6.4)	<0.001
		2014-2017	-1.1	(-3.9, 1.8)	<0.001
		2017-2019	30.4	(25.1, 35.8)	<0.001
mCRPC	Overall	2012-2017	15.4	(13.1, 17.6)	<0.001
		2017-2019	8.9	(2.4, 15.7)	0.02
	Non-Hispanic Black	2012-2019	11.8	(10.4, 13.3)	<0.001
	Hispanic	2012-2019	12.7	(10.2, 15.3)	<0.001
	Non-Hispanic White	2012-2017	16.2	(13.8, 18.5)	<0.001
		2017-2019	6.3	(-0.2, 13.1)	0.05

© 2024 Stock SR et al. *JAMA Network Open.*

eTable 10. Age-adjusted point prevalence (per 100K) and 95% confidence intervals by disease state and year.

Disease state	2012	2013	2014	2015	2016	2017	2018	2019	2020
**nmHSPC**	2523.54 (2508.73, 2538.35)	2508.06 (2493.56, 2522.56)	2470.67 (2456.69, 2484.64)	2440.94 (2427.28, 2454.60)	2409.13 (2395.78, 2422.49)	2392.72 (2379.63, 2405.80)	2424.66 (2411.71, 2437.61)	2443.23 (2430.49, 2455.98)	2411.60 (2399.06, 2424.14)
**Overall mHSPC**	116.75 (113.54, 119.97)	123.04 (119.81, 126.27)	133.59 (130.22, 136.95)	144.16 (140.77, 147.54)	156.34 (152.95, 159.72)	169.28 (165.84, 172.72)	191.59 (188.01, 195.16)	210.73 (207.09, 214.37)	227.31 (223.62, 231.01)
**De novo**	48.07 (45.86, 50.28)	49.04 (46.93, 51.16)	52.78 (50.54, 55.03)	55.06 (52.81, 57.32)	58.80 (56.52, 61.09)	62.05 (59.75, 64.36)	68.86 (66.50, 71.22)	75.37 (72.95, 77.79)	79.11 (76.70, 81.52)
**Recurrent**	68.68 (66.34, 71.02)	74.00 (71.55, 76.44)	80.80 (78.30, 83.31)	89.10 (86.57, 91.62)	97.53 (95.04, 100.02)	107.23 (104.67, 109.78)	122.73 (120.04, 125.41)	135.36 (132.65, 138.08)	148.20 (145.40, 151.00)
**nmCRPC**	14.17 (13.29, 15.04)	13.06 (12.29, 13.83)	12.44 (11.63, 13.26)	11.48 (10.75, 12.22)	11.47 (10.71, 12.23)	12.28 (11.41, 13.14)	14.17 (13.28, 15.07)	17.63 (16.65, 18.62)	16.44 (15.52, 17.37)
**mCRPC**	25.47 (24.08, 26.86)	30.20 (28.76, 31.64)	32.99 (31.64, 34.34)	37.52 (36.03, 39.02)	43.24 (41.65, 44.84)	53.06 (51.27, 54.86)	56.33 (54.51, 58.14)	60.84 (59.02, 62.67)	60.23 (58.46, 62.00)

© 2024 Stock SR et al. *JAMA Network Open.*

eTable 11. Age-adjusted point prevalence (per 100K) and 95% confidence intervals by race/ethnicity, disease state, and year.

Disease state	2012	2013	2014	2015	2016	2017	2018	2019	2020
**nmHSPC**									
Non-Hispanic White	2188.93 (2173.86, 2204.01)	2157.16 (2142.50, 2171.83)	2116.32 (2102.08, 2130.56)	2071.93 (2058.04, 2085.82)	2028.83 (2015.23, 2042.44)	2004.20 (1990.76, 2017.64)	2024.98 (2011.66, 2038.30)	2036.95 (2023.81, 2050.10)	2003.09 (1990.20, 2015.99)
Non-Hispanic Black	4077.55 (4032.26, 4122.84)	4111.79 (4067.07, 4156.51)	4096.36 (4052.79, 4139.93)	4111.01 (4067.77, 4154.24)	4117.14 (4074.43, 4159.86)	4135.71 (4093.62, 4177.79)	4199.54 (4157.80, 4241.28)	4259.45 (4217.98, 4300.92)	4194.83 (4154.20, 4235.46)
RR (vs. White)	1.86 (1.84, 1.89) P < 0.001	1.91 (1.88, 1.93) P < 0.001	1.94 (1.91, 1.96) P < 0.001	1.98 (1.96, 2.01) P < 0.001	2.03 (2.00, 2.05) P < 0.001	2.06 (2.04, 2.09) P < 0.001	2.07 (2.05, 2.10) P < 0.001	2.09 (2.07, 2.12) P < 0.001	2.09 (2.07, 2.12) P < 0.001
Hispanic	2242.75 (2188.94, 2296.56)	2215.46 (2163.14, 2267.78)	2185.28 (2135.00, 2235.57)	2163.49 (2115.60, 2211.38)	2130.07 (2084.30, 2175.83)	2103.91 (2060.57, 2147.25)	2133.89 (2091.04, 2176.75)	2149.07 (2107.19, 2190.96)	2137.66 (2096.10, 2179.23)
RR (vs. White)	1.02 (1.00, 1.05) P = 0.06	1.03 (1.00, 1.05) P = 0.03	1.03 (1.01, 1.06) P = 0.009	1.04 (1.02, 1.07) P < 0.001	1.05 (1.03, 1.07) P < 0.001	1.05 (1.03, 1.07) P < 0.001	1.05 (1.03, 1.08) P < 0.001	1.06 (1.03, 1.08) P < 0.001	1.07 (1.05, 1.09) P < 0.001
**Overall mHSPC**									
Non-Hispanic White	90.26 (86.93, 93.59)	94.15 (90.85, 97.45)	99.30 (96.03, 102.58)	106.46 (103.12, 109.80)	114.90 (111.63, 118.17)	123.93 (120.62, 127.25)	142.97 (139.43, 146.51)	159.83 (156.15, 163.51)	172.98 (169.26, 176.70)
Non-Hispanic Black	267.05 (256.19, 277.91)	281.15 (270.14, 292.16)	308.88 (297.22, 320.54)	333.22 (321.43, 345.01)	359.58 (347.54, 371.63)	384.73 (372.53, 396.92)	424.57 (412.06, 437.07)	459.48 (446.70, 472.26)	486.32 (473.42, 499.22)
RR (vs. White)	2.96 (2.80, 3.13) P < 0.001	2.99 (2.83, 3.15) P < 0.001	3.11 (2.96, 3.27) P < 0.001	3.13 (2.99, 3.28) P < 0.001	3.13 (2.99, 3.27) P < 0.001	3.10 (2.98, 3.24) P < 0.001	2.97 (2.86, 3.09) P < 0.001	2.87 (2.77, 2.98) P < 0.001	2.81 (2.72, 2.91) P < 0.001
Hispanic	127.18 (114.66, 139.70)	129.99 (118.39, 141.58)	138.77 (127.16, 150.38)	155.75 (143.65, 167.86)	165.64 (153.65, 177.62)	187.25 (174.37, 200.13)	211.99 (198.17, 225.82)	225.19 (211.99, 238.38)	236.49 (223.23, 249.75)
RR (vs. White)	1.41 (1.27, 1.57) P < 0.001	1.38 (1.25, 1.52) P < 0.001	1.40 (1.28, 1.53) P < 0.001	1.46 (1.35, 1.59) P < 0.001	1.44 (1.33, 1.56) P < 0.001	1.51 (1.40, 1.63) P < 0.001	1.48 (1.38, 1.59) P < 0.001	1.41 (1.32, 1.50) P < 0.001	1.37 (1.29, 1.45) P < 0.001

© 2024 Stock SR et al. *JAMA Network Open.*

Disease state	2012	2013	2014	2015	2016	2017	2018	2019	2020
**De novo mHSPC**									
Non-Hispanic White	36.12 (33.84, 38.40)	37.18 (34.98, 39.39)	39.46 (37.16, 41.76)	40.28 (37.94, 42.62)	41.87 (39.61, 44.13)	44.51 (42.23, 46.79)	51.30 (48.87, 53.72)	57.28 (54.73, 59.83)	60.23 (57.71, 62.75)
Non-Hispanic Black	111.56 (104.32, 118.79)	113.70 (106.63, 120.77)	118.63 (111.28, 125.98)	127.79 (120.31, 135.28)	136.48 (128.70, 144.26)	138.21 (130.52, 145.90)	146.00 (138.31, 153.70)	156.31 (148.48, 164.14)	162.21 (154.36, 170.07)
RR (vs. White)	3.09 (2.82, 3.38) P < 0.001	3.06 (2.81, 3.33) P < 0.001	3.01 (2.76, 3.27) P < 0.001	3.17 (2.92, 3.45) P < 0.001	3.26 (3.01, 3.53) P < 0.001	3.11 (2.88, 3.35) P < 0.001	2.85 (2.65, 3.06) P < 0.001	2.73 (2.55, 2.92) P < 0.001	2.69 (2.53, 2.87) P < 0.001
Hispanic	51.35 (42.97, 59.72)	47.24 (40.41, 54.08)	54.31 (46.89, 61.73)	55.57 (48.22, 62.91)	58.18 (51.06, 65.31)	65.28 (57.75, 72.82)	71.69 (63.45, 79.94)	78.15 (69.89, 86.42)	80.44 (72.25, 88.64)
RR (vs. White)	1.42 (1.19, 1.69) P < 0.001	1.27 (1.09, 1.49) P = 0.003	1.38 (1.19, 1.60) P < 0.001	1.38 (1.19, 1.59) P < 0.001	1.39 (1.22, 1.59) P < 0.001	1.47 (1.29, 1.66) P < 0.001	1.40 (1.23, 1.58) P < 0.001	1.36 (1.22, 1.53) P < 0.001	1.34 (1.20, 1.49) P < 0.001
**Recurrent mHSPC**									
Non-Hispanic White	54.14 (51.71, 56.58)	56.97 (54.52, 59.42)	59.84 (57.52, 62.17)	66.18 (63.80, 68.56)	73.03 (70.66, 75.40)	79.42 (77.02, 81.82)	91.67 (89.10, 94.25)	102.55 (99.89, 105.20)	112.75 (110.00, 115.49)
Non-Hispanic Black	155.49 (147.39, 163.59)	167.45 (159.01, 175.89)	190.25 (181.20, 199.30)	205.43 (196.32, 214.54)	223.10 (213.91, 232.30)	246.52 (237.05, 255.98)	278.56 (268.70, 288.42)	303.18 (293.07, 313.28)	324.11 (313.87, 334.34)
RR (vs. White)	2.87 (2.68, 3.08) P < 0.001	2.94 (2.75, 3.14) P < 0.001	3.18 (2.99, 3.38) P < 0.001	3.10 (2.93, 3.29) P < 0.001	3.05 (2.90, 3.22) P < 0.001	3.10 (2.96, 3.26) P < 0.001	3.04 (2.90, 3.18) P < 0.001	2.96 (2.83, 3.08) P < 0.001	2.87 (2.76, 2.99) P < 0.001
Hispanic	75.84 (66.53, 85.15)	82.74 (73.37, 92.11)	84.45 (75.53, 93.38)	100.18 (90.56, 109.80)	107.46 (97.82, 117.09)	121.96 (111.51, 132.42)	140.30 (129.20, 151.41)	147.03 (136.75, 157.32)	156.05 (145.62, 166.48)
RR (vs. White)	1.40 (1.23, 1.60) P < 0.001	1.45 (1.29, 1.64) P < 0.001	1.41 (1.26, 1.58) P < 0.001	1.51 (1.37, 1.68) P < 0.001	1.47 (1.34, 1.62) P < 0.001	1.54 (1.40, 1.68) P < 0.001	1.53 (1.41, 1.66) P < 0.001	1.43 (1.33, 1.54) P < 0.001	1.38 (1.29, 1.49) P < 0.001

© 2024 Stock SR et al. *JAMA Network Open.*

Disease state	2012	2013	2014	2015	2016	2017	2018	2019	2020
**nmCRPC**									
Non-Hispanic White	9.39 (8.57, 10.20)	8.42 (7.84, 9.00)	7.76 (7.12, 8.40)	7.09 (6.53, 7.65)	7.50 (6.78, 8.21)	7.51 (6.85, 8.17)	9.18 (8.42, 9.93)	12.55 (11.66, 13.44)	11.27 (10.50, 12.05)
Non-Hispanic Black	49.27 (44.92, 53.62)	45.46 (41.37, 49.56)	43.85 (39.76, 47.95)	39.35 (35.57, 43.13)	36.17 (32.58, 39.75)	39.37 (35.42, 43.32)	43.18 (39.20, 47.17)	47.64 (43.49, 51.78)	44.80 (40.88, 48.73)
RR (vs. White)	5.25 (4.64, 5.94) P < 0.001	5.40 (4.82, 6.05) P < 0.001	5.65 (4.99, 6.40) P < 0.001	5.55 (4.90, 6.28) P < 0.001	4.83 (4.21, 5.53) P < 0.001	5.24 (4.59, 5.99) P < 0.001	4.70 (4.16, 5.33) P < 0.001	3.80 (3.39, 4.25) P < 0.001	3.97 (3.56, 4.44) P < 0.001
Hispanic	24.09 (20.08, 28.11)	23.11 (18.55, 27.67)	21.54 (17.18, 25.91)	21.93 (17.14, 26.72)	20.01 (15.90, 24.11)	19.70 (15.73, 23.67)	19.38 (15.53, 23.23)	18.33 (14.66, 22.01)	17.33 (13.84, 20.83)
RR (vs. White)	2.57 (2.13, 3.10) P < 0.001	2.74 (2.23, 3.38) P < 0.001	2.78 (2.23, 3.46) P < 0.001	3.09 (2.45, 3.90) P < 0.001	2.67 (2.13, 3.35) P < 0.001	2.62 (2.11, 3.27) P < 0.001	2.11 (1.70, 2.62) P < 0.001	1.46 (1.18, 1.81) P < 0.001	1.54 (1.24, 1.90) P < 0.001
**mCRPC**									
Non-Hispanic White	19.73 (18.28, 21.18)	23.63 (22.15, 25.11)	26.25 (24.90, 27.60)	29.80 (28.34, 31.27)	34.86 (33.16, 36.57)	42.52 (40.63, 44.40)	44.30 (42.41, 46.18)	46.68 (44.84, 48.52)	46.08 (44.33, 47.83)
Non-Hispanic Black	64.38 (59.17, 69.60)	71.70 (66.41, 76.99)	77.30 (72.03, 82.58)	84.58 (78.89, 90.27)	95.19 (89.37, 101.00)	115.63 (109.13, 122.14)	124.11 (117.49, 130.73)	135.97 (129.22, 142.72)	134.07 (127.51, 140.62)
RR (vs. White)	3.26 (2.93, 3.64) P < 0.001	3.03 (2.75, 3.34) P < 0.001	2.95 (2.70, 3.21) P < 0.001	2.84 (2.61, 3.08) P < 0.001	2.73 (2.52, 2.95) P < 0.001	2.72 (2.53, 2.92) P < 0.001	2.80 (2.62, 3.00) P < 0.001	2.91 (2.73, 3.10) P < 0.001	2.91 (2.73, 3.10) P < 0.001
Hispanic	27.30 (22.87, 31.73)	34.08 (28.62, 39.54)	33.28 (27.97, 38.60)	37.01 (31.58, 42.44)	47.36 (41.00, 53.71)	54.11 (47.85, 60.37)	56.00 (49.96, 62.03)	63.51 (56.77, 70.24)	60.37 (54.04, 66.70)
RR (vs. White)	1.38 (1.16, 1.65) P < 0.001	1.44 (1.21, 1.71) P < 0.001	1.27 (1.07, 1.50) P = 0.006	1.24 (1.06, 1.45) P = 0.006	1.36 (1.18, 1.57) P < 0.001	1.27 (1.12, 1.44) P < 0.001	1.26 (1.13, 1.42) P < 0.001	1.36 (1.21, 1.52) P < 0.001	1.31 (1.17, 1.46) P < 0.001

© 2024 Stock SR et al. *JAMA Network Open.*

eFigure. Age-adjusted point prevalence (per 100K person-years) by race/ethnicity, disease state, and year. The dots represent the observed rates; the solid line represents model-based estimates obtained by the joinpoint analysis, with the Annual Percentage Change (APC) noted for each line segment.

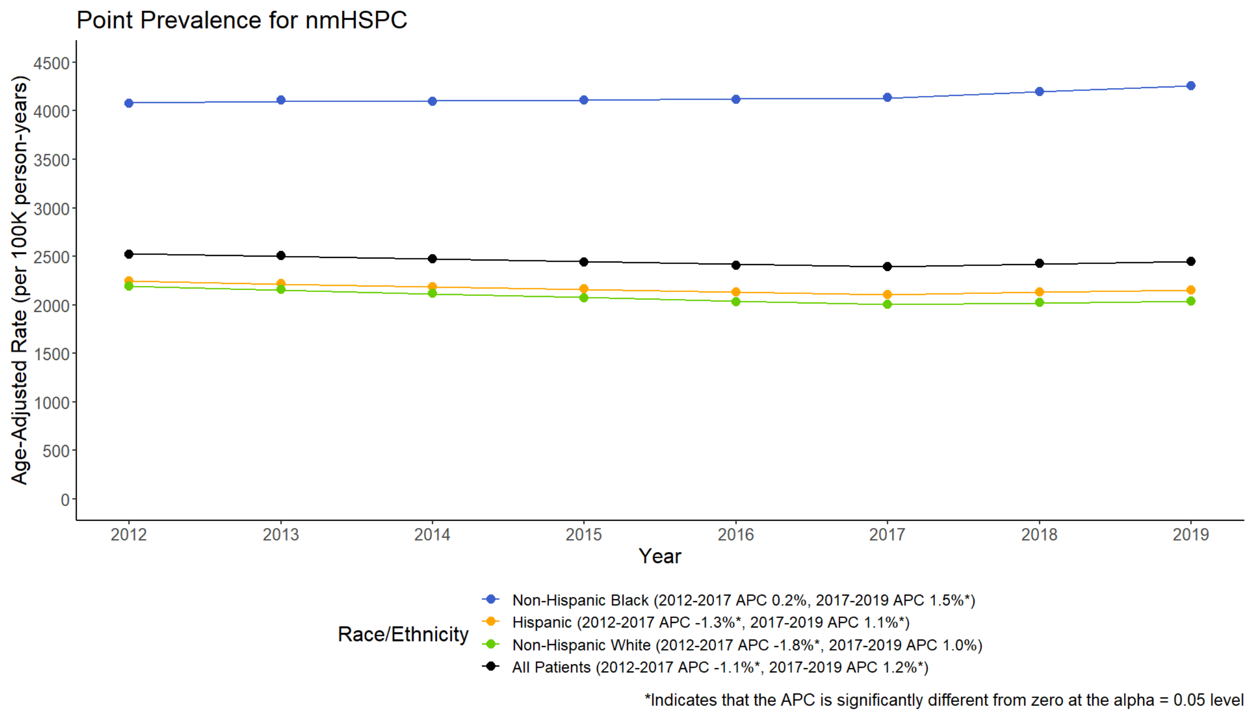

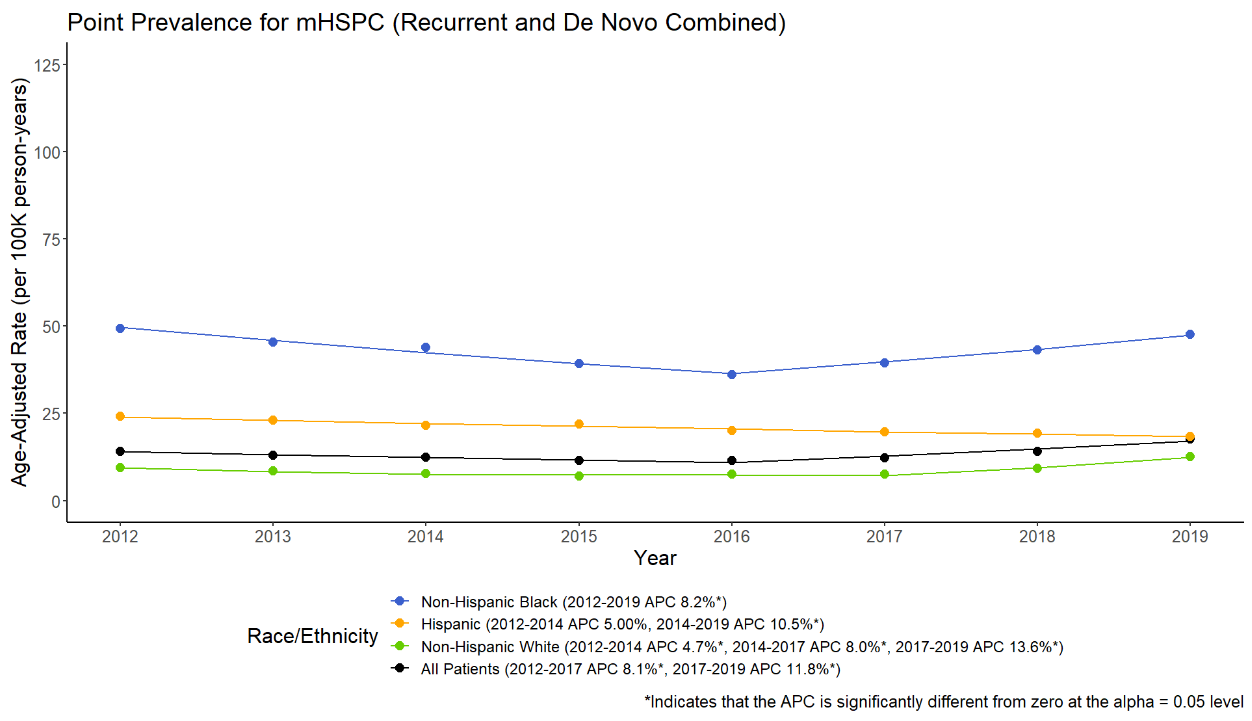

© 2024 Stock SR et al. *JAMA Network Open.*

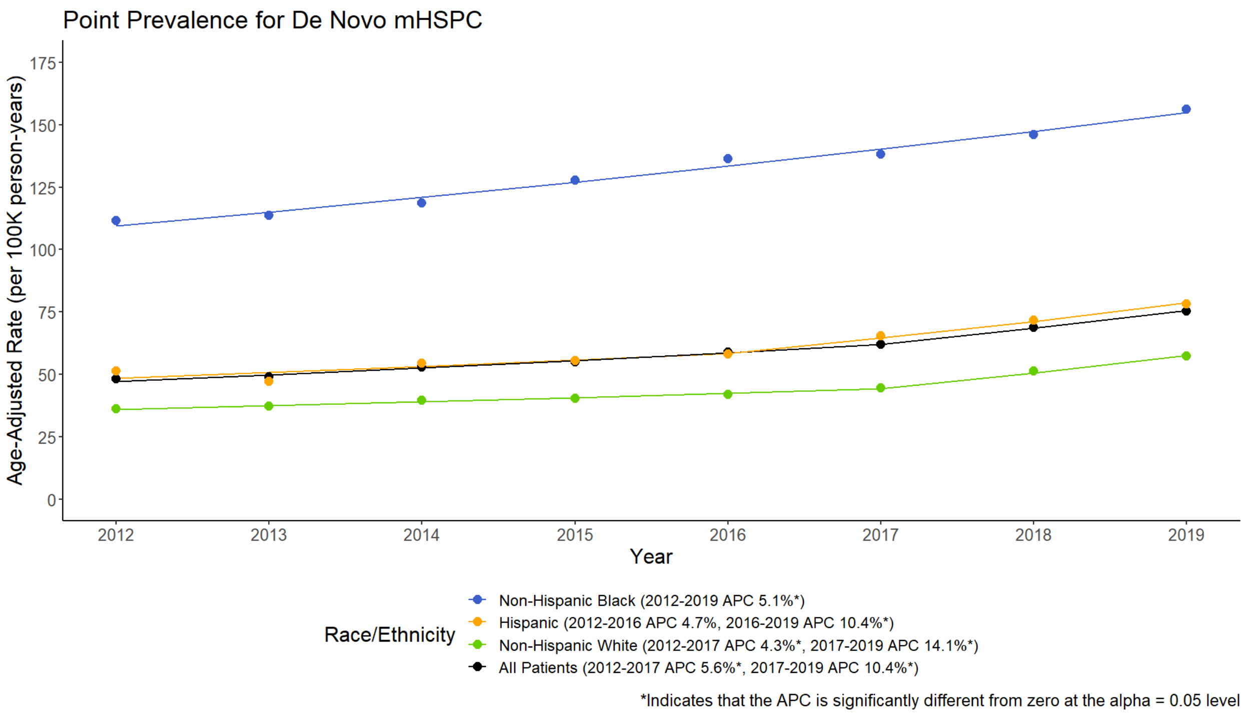

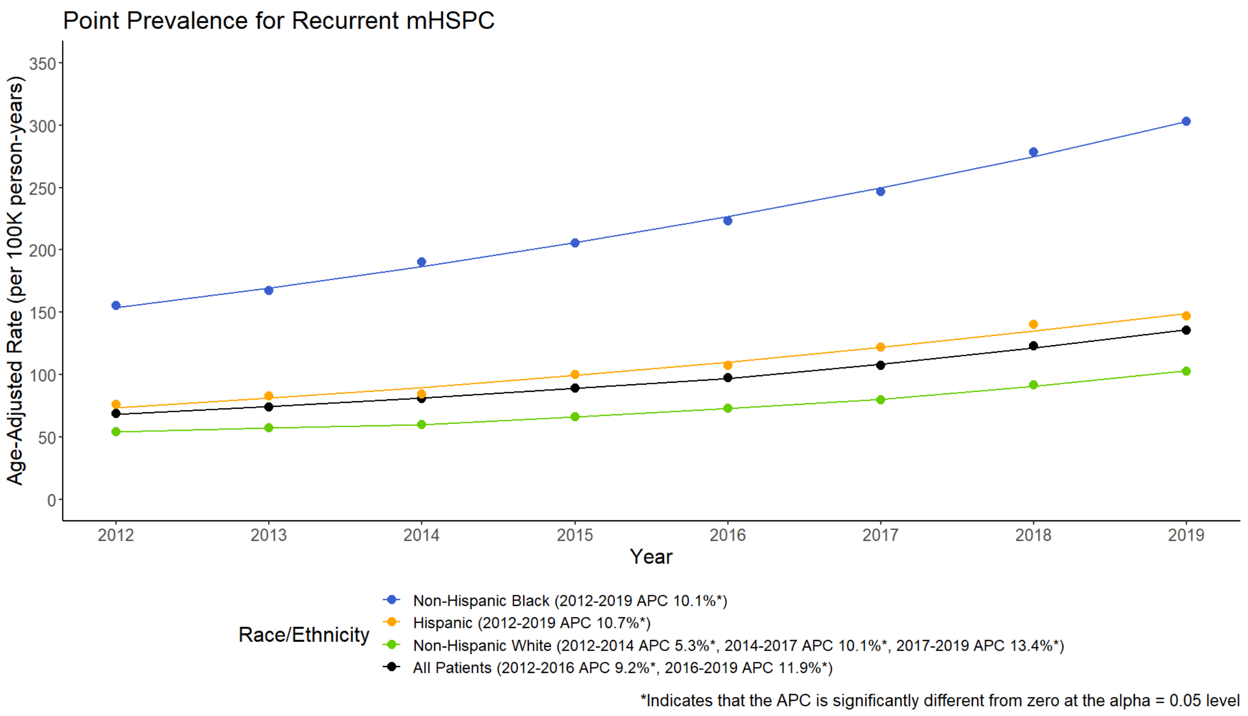

© 2024 Stock SR et al. *JAMA Network Open.*

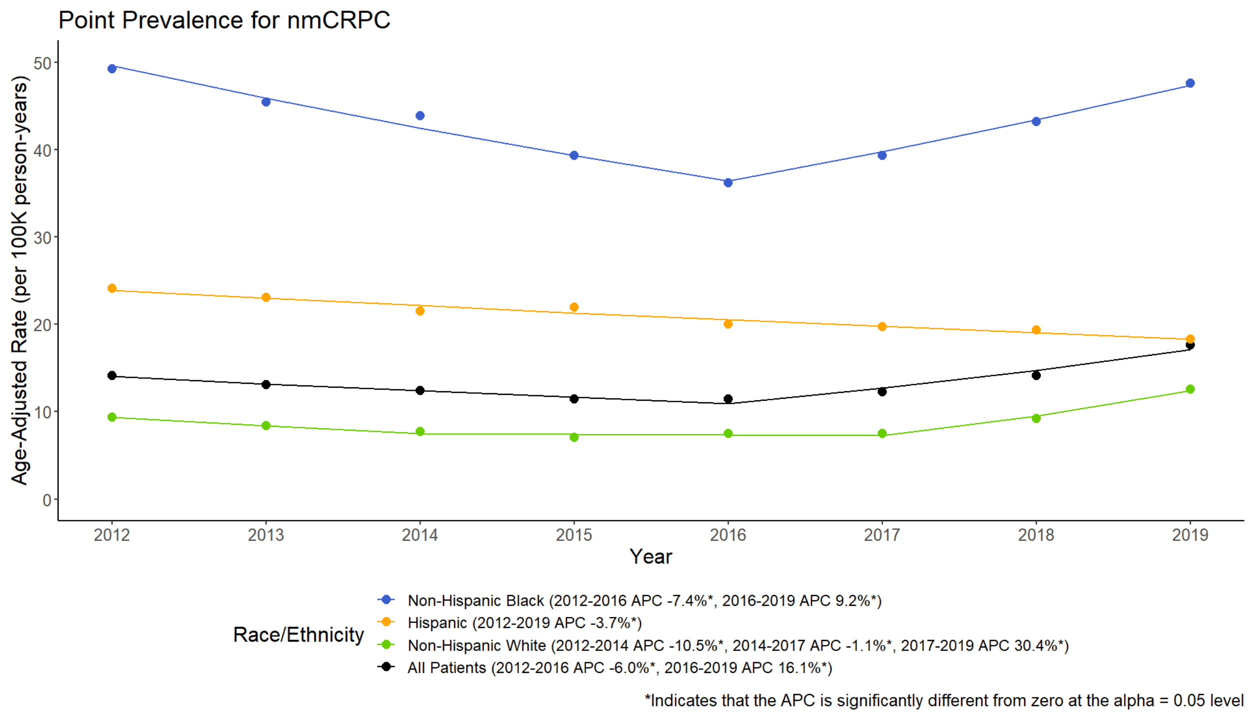

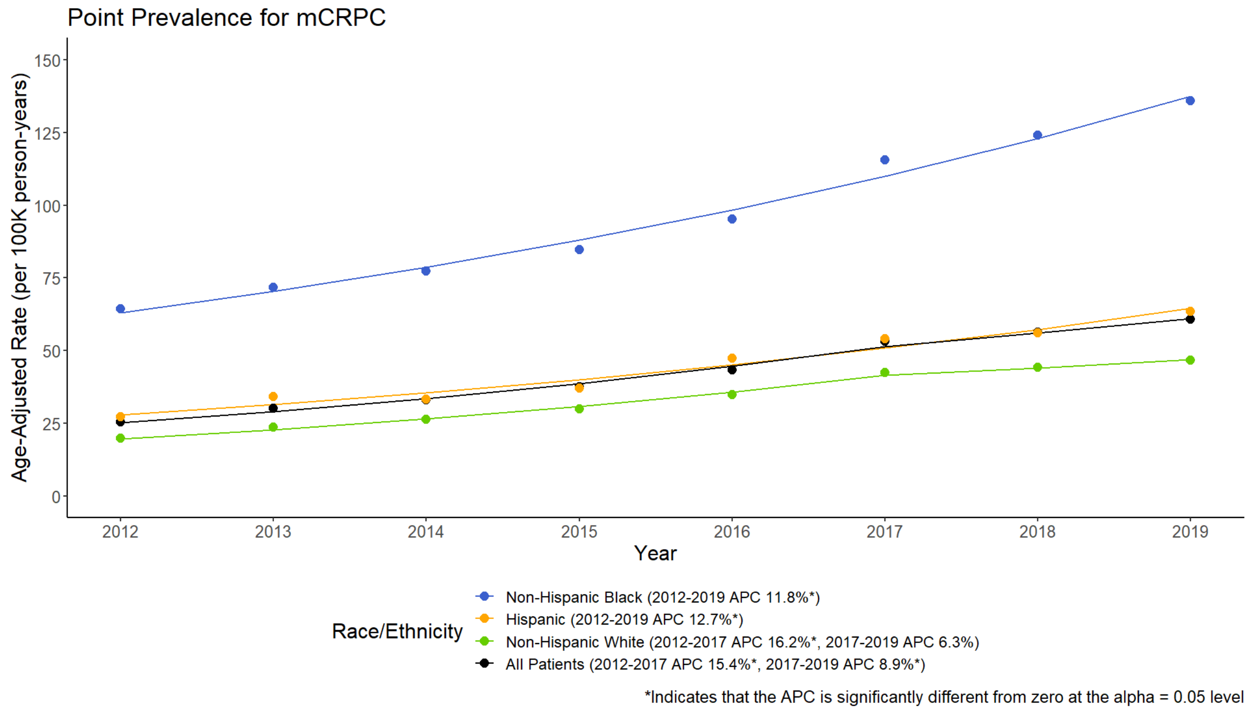

© 2024 Stock SR et al. *JAMA Network Open.*

eTable 12. Cumulative incidence estimates of time from nmHSPC diagnosis to disease progression or death as competing risks.

Estimates are provided among all patients, stratified by categories of race/ethnicity, stratified by categories of age, and stratified by combined categories of race/ethnicity and age.

		**Age**				
**Transition**	**Cumulative Incidence Estimate**	< 55	55-64	65-74	≥75	**All Patients**
mHSPC						
All Patients (N = 446,598)	2 year	0.03 (0.03, 0.03)	0.03 (0.03, 0.03)	0.03 (0.03, 0.03)	0.04 (0.04, 0.04)	0.03 (0.03, 0.03)
	3 year	0.04 (0.04, 0.05)	0.04 (0.04, 0.05)	0.04 (0.04, 0.04)	0.05 (0.05, 0.05)	0.04 (0.04, 0.04)
	5 year	0.06 (0.06, 0.07)	0.07 (0.07, 0.07)	0.06 (0.06, 0.06)	0.06 (0.06, 0.07)	0.06 (0.06, 0.07)
	8 year	0.09 (0.09, 0.1)	0.1 (0.09, 0.1)	0.09 (0.09, 0.09)	0.08 (0.08, 0.08)	0.09 (0.09, 0.09)
	10 year	0.11 (0.11, 0.12)	0.11 (0.11, 0.12)	0.1 (0.1, 0.1)	0.09 (0.09, 0.09)	0.1 (0.1, 0.1)
Black (N = 88,871)	2 year	0.03 (0.03, 0.04)	0.03 (0.03, 0.03)	0.04 (0.03, 0.04)	0.05 (0.05, 0.06)	0.04 (0.03, 0.04)
	3 year	0.04 (0.04, 0.05)	0.05 (0.04, 0.05)	0.05 (0.05, 0.05)	0.07 (0.06, 0.08)	0.05 (0.05, 0.05)
	5 year	0.06 (0.06, 0.07)	0.07 (0.07, 0.08)	0.08 (0.07, 0.08)	0.09 (0.09, 0.1)	0.08 (0.07, 0.08)
	8 year	0.1 (0.09, 0.1)	0.11 (0.1, 0.11)	0.11 (0.1, 0.11)	0.12 (0.11, 0.13)	0.11 (0.11, 0.11)
	10 year	0.12 (0.11, 0.13)	0.13 (0.12, 0.13)	0.12 (0.12, 0.13)	0.13 (0.13, 0.14)	0.13 (0.12, 0.13)
Hispanic (N = 19,998)	2 year	0.02 (0.01, 0.04)	0.03 (0.03, 0.04)	0.04 (0.03, 0.05)	0.05 (0.04, 0.06)	0.04 (0.04, 0.04)
	3 year	0.04 (0.02, 0.05)	0.05 (0.04, 0.05)	0.05 (0.05, 0.06)	0.06 (0.05, 0.07)	0.05 (0.05, 0.06)
	5 year	0.06 (0.04, 0.08)	0.07 (0.06, 0.08)	0.08 (0.07, 0.08)	0.09 (0.08, 0.1)	0.08 (0.07, 0.08)
	8 year	0.08 (0.05, 0.1)	0.09 (0.08, 0.1)	0.1 (0.09, 0.11)	0.12 (0.11, 0.13)	0.1 (0.1, 0.11)
	10 year	0.1 (0.07, 0.12)	0.11 (0.1, 0.12)	0.12 (0.11, 0.13)	0.13 (0.12, 0.15)	0.12 (0.11, 0.13)
White (N = 324,434)	2 year	0.03 (0.03, 0.04)	0.03 (0.03, 0.03)	0.03 (0.03, 0.03)	0.03 (0.03, 0.03)	0.03 (0.03, 0.03)
	3 year	0.04 (0.04, 0.05)	0.04 (0.04, 0.04)	0.04 (0.04, 0.04)	0.04 (0.04, 0.04)	0.04 (0.04, 0.04)
	5 year	0.06 (0.06, 0.07)	0.06 (0.06, 0.07)	0.06 (0.06, 0.06)	0.06 (0.05, 0.06)	0.06 (0.06, 0.06)
	8 year	0.09 (0.08, 0.1)	0.09 (0.09, 0.09)	0.08 (0.08, 0.08)	0.07 (0.07, 0.07)	0.08 (0.08, 0.08)
	10 year	0.11 (0.1, 0.12)	0.11 (0.1, 0.11)	0.09 (0.09, 0.09)	0.08 (0.08, 0.08)	0.09 (0.09, 0.09)

© 2024 Stock SR et al. *JAMA Network Open.*

		Age				
**Transition**	**Cumulative Incidence Estimate**	< 55	55-64	65-74	≥75	**All Patients**
nmCRPC						
All Patients (N = 446,598)	2 year	0 (0, 0)	0.01 (0, 0.01)	0 (0, 0.01)	0.01 (0.01, 0.01)	0.01 (0.01, 0.01)
	3 year	0 (0, 0.01)	0.01 (0.01, 0.01)	0.01 (0.01, 0.01)	0.02 (0.02, 0.02)	0.01 (0.01, 0.01)
	5 year	0.01 (0.01, 0.01)	0.01 (0.01, 0.01)	0.01 (0.01, 0.01)	0.02 (0.02, 0.02)	0.01 (0.01, 0.01)
	8 year	0.01 (0.01, 0.01)	0.02 (0.02, 0.02)	0.01 (0.01, 0.02)	0.03 (0.03, 0.03)	0.02 (0.02, 0.02)
	10 year	0.02 (0.01, 0.02)	0.02 (0.02, 0.02)	0.02 (0.02, 0.02)	0.03 (0.03, 0.03)	0.02 (0.02, 0.02)
Black (N = 88,871)	2 year	0 (0, 0.01)	0.01 (0.01, 0.01)	0.01 (0.01, 0.01)	0.02 (0.02, 0.02)	0.01 (0.01, 0.01)
	3 year	0.01 (0, 0.01)	0.01 (0.01, 0.01)	0.01 (0.01, 0.01)	0.03 (0.02, 0.03)	0.01 (0.01, 0.01)
	5 year	0.01 (0.01, 0.01)	0.01 (0.01, 0.01)	0.01 (0.01, 0.02)	0.04 (0.03, 0.04)	0.02 (0.01, 0.02)
	8 year	0.01 (0.01, 0.01)	0.02 (0.02, 0.02)	0.02 (0.02, 0.02)	0.05 (0.04, 0.05)	0.02 (0.02, 0.02)
	10 year	0.01 (0.01, 0.02)	0.02 (0.02, 0.02)	0.02 (0.02, 0.03)	0.05 (0.05, 0.06)	0.03 (0.02, 0.03)
Hispanic (N = 19,998)	2 year	0.01 (0, 0.01)	0 (0, 0.01)	0.01 (0, 0.01)	0.01 (0.01, 0.02)	0.01 (0.01, 0.01)
	3 year	0.01 (0, 0.01)	0.01 (0, 0.01)	0.01 (0.01, 0.01)	0.02 (0.02, 0.03)	0.01 (0.01, 0.01)
	5 year	0.01 (0, 0.02)	0.01 (0.01, 0.01)	0.01 (0.01, 0.01)	0.03 (0.02, 0.04)	0.02 (0.01, 0.02)
	8 year	0.02 (0.01, 0.03)	0.01 (0.01, 0.02)	0.02 (0.01, 0.02)	0.04 (0.03, 0.05)	0.02 (0.02, 0.03)
	10 year	0.02 (0.01, 0.03)	0.02 (0.02, 0.03)	0.02 (0.02, 0.03)	0.04 (0.03, 0.05)	0.03 (0.02, 0.03)
White (N = 324,434)	2 year	0 (0, 0)	0 (0, 0.01)	0 (0, 0)	0.01 (0.01, 0.01)	0.01 (0.01, 0.01)
	3 year	0 (0, 0.01)	0.01 (0.01, 0.01)	0.01 (0.01, 0.01)	0.01 (0.01, 0.02)	0.01 (0.01, 0.01)
	5 year	0.01 (0, 0.01)	0.01 (0.01, 0.01)	0.01 (0.01, 0.01)	0.02 (0.02, 0.02)	0.01 (0.01, 0.01)
	8 year	0.01 (0.01, 0.01)	0.01 (0.01, 0.02)	0.01 (0.01, 0.01)	0.02 (0.02, 0.03)	0.02 (0.02, 0.02)
	10 year	0.02 (0.01, 0.02)	0.02 (0.02, 0.02)	0.02 (0.01, 0.02)	0.03 (0.03, 0.03)	0.02 (0.02, 0.02)

© 2024 Stock SR et al. *JAMA Network Open.*

		Age				
**Transition**	**Cumulative Incidence Estimate**	< 55	55-64	65-74	≥75	**All Patients**
mCRPC						
All Patients (N = 446,598)	2 year	0 (0, 0)	0 (0, 0)	0 (0, 0)	0.01 (0.01, 0.01)	0 (0, 0.01)
	3 year	0 (0, 0)	0 (0, 0)	0.01 (0.01, 0.01)	0.01 (0.01, 0.01)	0.01 (0.01, 0.01)
	5 year	0 (0, 0)	0.01 (0.01, 0.01)	0.01 (0.01, 0.01)	0.01 (0.01, 0.02)	0.01 (0.01, 0.01)
	8 year	0 (0, 0.01)	0.01 (0.01, 0.01)	0.01 (0.01, 0.01)	0.02 (0.02, 0.02)	0.01 (0.01, 0.01)
	10 year	0.01 (0, 0.01)	0.01 (0.01, 0.01)	0.01 (0.01, 0.01)	0.02 (0.02, 0.02)	0.01 (0.01, 0.01)
Black (N = 88,871)	2 year	0 (0, 0)	0 (0, 0)	0 (0, 0)	0.01 (0, 0.01)	0 (0, 0)
	3 year	0 (0, 0)	0 (0, 0)	0 (0, 0.01)	0.01 (0.01, 0.01)	0 (0, 0)
	5 year	0 (0, 0)	0 (0, 0.01)	0.01 (0, 0.01)	0.01 (0.01, 0.01)	0.01 (0, 0.01)
	8 year	0 (0, 0.01)	0.01 (0.01, 0.01)	0.01 (0.01, 0.01)	0.01 (0.01, 0.02)	0.01 (0.01, 0.01)
	10 year	0.01 (0, 0.01)	0.01 (0.01, 0.01)	0.01 (0.01, 0.01)	0.01 (0.01, 0.02)	0.01 (0.01, 0.01)
Hispanic (N = 19,998)	2 year	0 (0, 0)	0 (0, 0.01)	0 (0, 0)	0.01 (0, 0.01)	0 (0, 0)
	3 year	0 (0, 0.01)	0 (0, 0.01)	0 (0, 0.01)	0.01 (0, 0.01)	0 (0, 0.01)
	5 year	0 (0, 0.01)	0.01 (0, 0.01)	0 (0, 0.01)	0.01 (0, 0.01)	0.01 (0, 0.01)
	8 year	0 (0, 0.01)	0.01 (0, 0.01)	0.01 (0, 0.01)	0.01 (0.01, 0.01)	0.01 (0.01, 0.01)
	10 year	0 (0, 0.01)	0.01 (0, 0.01)	0.01 (0.01, 0.01)	0.01 (0.01, 0.01)	0.01 (0.01, 0.01)
White (N = 324,434)	2 year	0 (0, 0.01)	0 (0, 0)	0 (0, 0.01)	0.01 (0.01, 0.01)	0.01 (0.01, 0.01)
	3 year	0 (0, 0.01)	0 (0, 0.01)	0.01 (0.01, 0.01)	0.01 (0.01, 0.01)	0.01 (0.01, 0.01)
	5 year	0 (0, 0.01)	0.01 (0.01, 0.01)	0.01 (0.01, 0.01)	0.02 (0.01, 0.02)	0.01 (0.01, 0.01)
	8 year	0.01 (0, 0.01)	0.01 (0.01, 0.01)	0.01 (0.01, 0.01)	0.02 (0.02, 0.02)	0.01 (0.01, 0.01)
	10 year	0.01 (0, 0.01)	0.01 (0.01, 0.01)	0.01 (0.01, 0.01)	0.02 (0.02, 0.02)	0.01 (0.01, 0.02)

© 2024 Stock SR et al. *JAMA Network Open.*

		Age				
**Transition**	**Cumulative Incidence Estimate**	< 55	55-64	65-74	≥75	**All Patients**
Death						
All Patients (N = 446,598)	2 year	0.01 (0.01, 0.01)	0.01 (0.01, 0.01)	0.02 (0.02, 0.02)	0.09 (0.09, 0.1)	0.04 (0.03, 0.04)
	3 year	0.02 (0.01, 0.02)	0.02 (0.02, 0.02)	0.03 (0.03, 0.04)	0.14 (0.13, 0.14)	0.05 (0.05, 0.06)
	5 year	0.03 (0.02, 0.03)	0.04 (0.04, 0.04)	0.06 (0.06, 0.07)	0.22 (0.22, 0.23)	0.09 (0.09, 0.09)
	8 year	0.05 (0.04, 0.05)	0.07 (0.07, 0.07)	0.12 (0.11, 0.12)	0.36 (0.35, 0.36)	0.16 (0.16, 0.16)
	10 year	0.06 (0.06, 0.07)	0.09 (0.09, 0.1)	0.16 (0.16, 0.16)	0.44 (0.44, 0.45)	0.21 (0.21, 0.21)
Black (N = 88,871)	2 year	0.01 (0.01, 0.01)	0.02 (0.01, 0.02)	0.03 (0.02, 0.03)	0.11 (0.11, 0.12)	0.03 (0.03, 0.03)
	3 year	0.02 (0.01, 0.02)	0.02 (0.02, 0.03)	0.04 (0.04, 0.05)	0.17 (0.16, 0.18)	0.05 (0.05, 0.05)
	5 year	0.03 (0.02, 0.03)	0.04 (0.04, 0.05)	0.07 (0.07, 0.08)	0.26 (0.25, 0.27)	0.08 (0.08, 0.08)
	8 year	0.05 (0.04, 0.05)	0.08 (0.07, 0.08)	0.13 (0.13, 0.14)	0.37 (0.36, 0.39)	0.13 (0.13, 0.13)
	10 year	0.06 (0.05, 0.07)	0.1 (0.1, 0.11)	0.18 (0.17, 0.18)	0.45 (0.44, 0.47)	0.17 (0.17, 0.17)
Hispanic (N = 19,998)	2 year	0.01 (0, 0.01)	0.01 (0, 0.01)	0.01 (0.01, 0.02)	0.1 (0.09, 0.11)	0.03 (0.03, 0.04)
	3 year	0.01 (0, 0.02)	0.01 (0.01, 0.02)	0.02 (0.02, 0.03)	0.13 (0.12, 0.15)	0.05 (0.04, 0.05)
	5 year	0.02 (0.01, 0.03)	0.03 (0.02, 0.03)	0.04 (0.04, 0.05)	0.21 (0.2, 0.23)	0.08 (0.07, 0.08)
	8 year	0.04 (0.02, 0.05)	0.05 (0.04, 0.06)	0.09 (0.08, 0.09)	0.33 (0.31, 0.34)	0.13 (0.13, 0.14)
	10 year	0.05 (0.03, 0.07)	0.07 (0.06, 0.07)	0.12 (0.11, 0.13)	0.4 (0.38, 0.42)	0.17 (0.16, 0.18)
White (N = 324,434)	2 year	0.01 (0.01, 0.01)	0.01 (0.01, 0.01)	0.02 (0.02, 0.02)	0.09 (0.09, 0.09)	0.04 (0.04, 0.04)
	3 year	0.02 (0.01, 0.02)	0.02 (0.02, 0.02)	0.03 (0.03, 0.03)	0.13 (0.13, 0.14)	0.06 (0.06, 0.06)
	5 year	0.03 (0.02, 0.03)	0.04 (0.04, 0.04)	0.06 (0.06, 0.06)	0.22 (0.22, 0.22)	0.1 (0.1, 0.1)
	8 year	0.05 (0.04, 0.06)	0.07 (0.07, 0.07)	0.11 (0.11, 0.12)	0.35 (0.35, 0.36)	0.17 (0.17, 0.17)
	10 year	0.07 (0.06, 0.08)	0.09 (0.09, 0.09)	0.16 (0.15, 0.16)	0.45 (0.44, 0.45)	0.22 (0.22, 0.22)

© 2024 Stock SR et al. *JAMA Network Open.*

eTable 13. Cumulative incidence estimates of time from de novo mHSPC diagnosis to mCRPC disease progression or death as competing risks.

Estimates are provided among all patients, stratified by categories of race/ethnicity, stratified by categories of age, and stratified by combined categories of race/ethnicity and age.

		**Age**				
**Transition**	**Cumulative Incidence Estimate**	< 55	55-64	65-74	≥75	**All Patients**
mCRPC						
All Patients (N = 18,201)	2 year	0.26 (0.22, 0.31)	0.27 (0.25, 0.29)	0.25 (0.24, 0.26)	0.29 (0.28, 0.3)	0.27 (0.26, 0.28)
	3 year	0.29 (0.24, 0.33)	0.31 (0.29, 0.33)	0.29 (0.28, 0.3)	0.34 (0.33, 0.35)	0.31 (0.31, 0.32)
	5 year	0.35 (0.3, 0.39)	0.35 (0.33, 0.37)	0.33 (0.32, 0.34)	0.37 (0.36, 0.38)	0.35 (0.34, 0.36)
	8 year	0.39 (0.34, 0.43)	0.39 (0.37, 0.4)	0.37 (0.35, 0.38)	0.39 (0.37, 0.4)	0.38 (0.37, 0.39)
	10 year	0.4 (0.35, 0.45)	0.4 (0.38, 0.42)	0.38 (0.36, 0.39)	0.39 (0.38, 0.4)	0.39 (0.38, 0.4)
Black (N = 4,924)	2 year	0.25 (0.19, 0.3)	0.27 (0.25, 0.3)	0.24 (0.22, 0.27)	0.34 (0.31, 0.37)	0.28 (0.26, 0.29)
	3 year	0.27 (0.21, 0.33)	0.31 (0.28, 0.33)	0.28 (0.26, 0.3)	0.38 (0.35, 0.41)	0.31 (0.3, 0.33)
	5 year	0.33 (0.26, 0.39)	0.34 (0.31, 0.37)	0.31 (0.28, 0.33)	0.4 (0.37, 0.43)	0.34 (0.33, 0.36)
	8 year	0.38 (0.31, 0.44)	0.38 (0.35, 0.4)	0.35 (0.32, 0.38)	0.43 (0.4, 0.46)	0.38 (0.36, 0.4)
	10 year	0.4 (0.33, 0.46)	0.39 (0.36, 0.41)	0.37 (0.34, 0.4)	0.43 (0.4, 0.46)	0.39 (0.38, 0.41)
Hispanic (N = 1,024)	2 year	0.06 (0, 0.16)	0.21 (0.15, 0.27)	0.21 (0.16, 0.26)	0.28 (0.24, 0.33)	0.24 (0.21, 0.27)
	3 year	0.11 (0, 0.25)	0.26 (0.19, 0.32)	0.24 (0.19, 0.28)	0.33 (0.28, 0.37)	0.28 (0.24, 0.3)
	5 year	0.23 (0, 0.4)	0.3 (0.23, 0.37)	0.28 (0.23, 0.33)	0.36 (0.31, 0.41)	0.32 (0.28, 0.35)
	8 year	0.28 (0.03, 0.47)	0.38 (0.3, 0.45)	0.32 (0.26, 0.38)	0.39 (0.33, 0.43)	0.36 (0.33, 0.4)
	10 year	0.36 (0.07, 0.55)	0.41 (0.33, 0.49)	0.33 (0.27, 0.39)	0.39 (0.34, 0.44)	0.38 (0.34, 0.41)
White (N = 11,670)	2 year	0.29 (0.22, 0.36)	0.28 (0.25, 0.3)	0.26 (0.24, 0.27)	0.28 (0.27, 0.29)	0.27 (0.26, 0.28)
	3 year	0.32 (0.24, 0.38)	0.32 (0.3, 0.34)	0.3 (0.29, 0.32)	0.33 (0.32, 0.35)	0.32 (0.31, 0.33)
	5 year	0.37 (0.3, 0.44)	0.37 (0.34, 0.39)	0.34 (0.33, 0.36)	0.36 (0.35, 0.38)	0.36 (0.35, 0.37)
	8 year	0.4 (0.32, 0.47)	0.4 (0.37, 0.42)	0.38 (0.36, 0.39)	0.38 (0.36, 0.39)	0.38 (0.37, 0.39)
	10 year	0.4 (0.33, 0.47)	0.41 (0.38, 0.43)	0.39 (0.37, 0.4)	0.38 (0.37, 0.4)	0.39 (0.38, 0.4)

© 2024 Stock SR et al. *JAMA Network Open.*

		Age				
**Transition**	**Cumulative Incidence Estimate**	< 55	55-64	65-74	≥75	**All Patients**
Death						
All Patients (N = 18,201)	2 year	0.05 (0.03, 0.07)	0.07 (0.06, 0.08)	0.11 (0.1, 0.11)	0.34 (0.33, 0.35)	0.18 (0.17, 0.19)
	3 year	0.06 (0.03, 0.08)	0.09 (0.08, 0.1)	0.13 (0.12, 0.14)	0.38 (0.36, 0.39)	0.2 (0.2, 0.21)
	5 year	0.09 (0.06, 0.11)	0.11 (0.1, 0.12)	0.16 (0.15, 0.17)	0.42 (0.41, 0.43)	0.24 (0.23, 0.24)
	8 year	0.1 (0.07, 0.13)	0.14 (0.13, 0.15)	0.19 (0.18, 0.2)	0.46 (0.45, 0.47)	0.27 (0.26, 0.28)
	10 year	0.13 (0.09, 0.16)	0.16 (0.14, 0.17)	0.21 (0.2, 0.23)	0.49 (0.47, 0.5)	0.29 (0.28, 0.3)
Black (N = 4,924)	2 year	0.03 (0.01, 0.06)	0.06 (0.04, 0.07)	0.1 (0.09, 0.12)	0.36 (0.33, 0.39)	0.15 (0.13, 0.16)
	3 year	0.04 (0.01, 0.06)	0.07 (0.06, 0.09)	0.13 (0.11, 0.15)	0.39 (0.36, 0.42)	0.17 (0.16, 0.18)
	5 year	0.06 (0.03, 0.09)	0.1 (0.08, 0.11)	0.15 (0.14, 0.17)	0.43 (0.4, 0.46)	0.2 (0.18, 0.21)
	8 year	0.08 (0.04, 0.11)	0.13 (0.11, 0.14)	0.19 (0.17, 0.21)	0.49 (0.45, 0.52)	0.23 (0.22, 0.25)
	10 year	0.1 (0.05, 0.14)	0.14 (0.12, 0.16)	0.21 (0.18, 0.23)	0.51 (0.48, 0.54)	0.25 (0.24, 0.27)
Hispanic (N = 1,024)	2 year	0.11 (0, 0.25)	0.08 (0.04, 0.12)	0.11 (0.07, 0.14)	0.36 (0.31, 0.41)	0.2 (0.18, 0.23)
	3 year	0.18 (0, 0.34)	0.08 (0.04, 0.12)	0.13 (0.09, 0.17)	0.39 (0.34, 0.44)	0.22 (0.19, 0.25)
	5 year	0.18 (0, 0.34)	0.1 (0.06, 0.15)	0.14 (0.1, 0.18)	0.44 (0.39, 0.49)	0.25 (0.22, 0.28)
	8 year	0.18 (0, 0.34)	0.11 (0.06, 0.16)	0.15 (0.11, 0.2)	0.48 (0.42, 0.53)	0.27 (0.24, 0.3)
	10 year	0.18 (0, 0.34)	0.13 (0.08, 0.18)	0.2 (0.14, 0.25)	0.51 (0.45, 0.56)	0.31 (0.27, 0.34)
White (N = 11,670)	2 year	0.07 (0.03, 0.1)	0.08 (0.07, 0.1)	0.11 (0.1, 0.12)	0.34 (0.32, 0.35)	0.19 (0.19, 0.2)
	3 year	0.07 (0.03, 0.11)	0.1 (0.08, 0.11)	0.13 (0.12, 0.14)	0.37 (0.35, 0.38)	0.22 (0.21, 0.23)
	5 year	0.11 (0.06, 0.16)	0.12 (0.11, 0.14)	0.16 (0.15, 0.17)	0.41 (0.4, 0.43)	0.25 (0.24, 0.26)
	8 year	0.13 (0.07, 0.18)	0.16 (0.14, 0.17)	0.2 (0.18, 0.21)	0.45 (0.43, 0.47)	0.29 (0.28, 0.3)
	10 year	0.17 (0.11, 0.23)	0.17 (0.15, 0.19)	0.21 (0.2, 0.23)	0.47 (0.46, 0.49)	0.3 (0.29, 0.31)

© 2024 Stock SR et al. *JAMA Network Open.*

eTable 14. Cumulative incidence estimates of time from recurrent mHSPC diagnosis to mCRPC disease progression or death as competing risks.

Estimates are provided among all patients, stratified by categories of race/ethnicity, stratified by categories of age, and stratified by combined categories of race/ethnicity and age.

		Age				
**Transition**	**Cumulative Incidence Estimate**	< 55	55-64	65-74	≥75	**All Patients**
mCRPC						
All Patients (N = 36,726)	2 year	0.12 (0.08, 0.15)	0.15 (0.14, 0.16)	0.15 (0.14, 0.16)	0.18 (0.17, 0.19)	0.16 (0.16, 0.17)
	3 year	0.14 (0.11, 0.18)	0.19 (0.18, 0.2)	0.18 (0.17, 0.19)	0.21 (0.2, 0.22)	0.19 (0.19, 0.2)
	5 year	0.17 (0.13, 0.21)	0.23 (0.21, 0.24)	0.22 (0.21, 0.23)	0.23 (0.23, 0.24)	0.23 (0.22, 0.23)
	8 year	0.22 (0.18, 0.26)	0.26 (0.25, 0.27)	0.25 (0.24, 0.26)	0.26 (0.25, 0.27)	0.26 (0.25, 0.26)
	10 year	0.23 (0.19, 0.28)	0.28 (0.27, 0.3)	0.27 (0.26, 0.28)	0.26 (0.26, 0.27)	0.27 (0.27, 0.28)
Black (N = 9,581)	2 year	0.11 (0.06, 0.15)	0.16 (0.14, 0.17)	0.15 (0.14, 0.16)	0.18 (0.16, 0.19)	0.16 (0.15, 0.17)
	3 year	0.13 (0.08, 0.17)	0.19 (0.18, 0.21)	0.18 (0.17, 0.2)	0.2 (0.19, 0.22)	0.19 (0.18, 0.2)
	5 year	0.17 (0.11, 0.22)	0.23 (0.21, 0.25)	0.23 (0.21, 0.24)	0.23 (0.21, 0.25)	0.23 (0.22, 0.24)
	8 year	0.23 (0.17, 0.28)	0.27 (0.24, 0.29)	0.27 (0.25, 0.29)	0.26 (0.24, 0.28)	0.27 (0.25, 0.28)
	10 year	0.25 (0.18, 0.31)	0.29 (0.26, 0.31)	0.29 (0.27, 0.32)	0.28 (0.26, 0.3)	0.29 (0.27, 0.3)
Hispanic (N = 2,040)	2 year	0.11 (0, 0.23)	0.11 (0.07, 0.16)	0.11 (0.09, 0.14)	0.15 (0.13, 0.18)	0.13 (0.12, 0.15)
	3 year	0.17 (0, 0.32)	0.15 (0.1, 0.2)	0.14 (0.11, 0.17)	0.19 (0.16, 0.22)	0.17 (0.15, 0.19)
	5 year	0.17 (0, 0.32)	0.2 (0.14, 0.25)	0.19 (0.15, 0.22)	0.22 (0.19, 0.25)	0.21 (0.18, 0.23)
	8 year	0.17 (0, 0.32)	0.21 (0.15, 0.27)	0.24 (0.19, 0.28)	0.25 (0.21, 0.28)	0.23 (0.21, 0.26)
	10 year	0.17 (0, 0.32)	0.22 (0.16, 0.28)	0.24 (0.2, 0.29)	0.26 (0.22, 0.29)	0.24 (0.22, 0.27)
White (N = 23,872)	2 year	0.14 (0.08, 0.2)	0.15 (0.14, 0.17)	0.15 (0.14, 0.16)	0.18 (0.18, 0.19)	0.17 (0.16, 0.17)
	3 year	0.18 (0.11, 0.24)	0.18 (0.17, 0.2)	0.18 (0.17, 0.19)	0.21 (0.2, 0.22)	0.19 (0.19, 0.2)
	5 year	0.2 (0.13, 0.26)	0.23 (0.21, 0.24)	0.22 (0.21, 0.23)	0.24 (0.23, 0.25)	0.23 (0.22, 0.23)
	8 year	0.23 (0.16, 0.3)	0.26 (0.24, 0.28)	0.25 (0.24, 0.26)	0.26 (0.25, 0.27)	0.26 (0.25, 0.26)
	10 year	0.24 (0.16, 0.31)	0.28 (0.26, 0.31)	0.27 (0.26, 0.28)	0.26 (0.25, 0.27)	0.27 (0.26, 0.28)

© 2024 Stock SR et al. *JAMA Network Open.*

		Age				
**Transition**	**Cumulative Incidence Estimate**	< 55	55-64	65-74	≥75	**All Patients**
Death						
All Patients (N = 36,726)	2 year	0.02 (0.01, 0.04)	0.06 (0.05, 0.07)	0.08 (0.08, 0.09)	0.25 (0.25, 0.26)	0.14 (0.14, 0.15)
	3 year	0.03 (0.02, 0.05)	0.07 (0.06, 0.08)	0.11 (0.1, 0.11)	0.3 (0.29, 0.31)	0.18 (0.17, 0.18)
	5 year	0.05 (0.02, 0.07)	0.1 (0.09, 0.11)	0.14 (0.13, 0.15)	0.38 (0.37, 0.39)	0.22 (0.22, 0.23)
	8 year	0.06 (0.04, 0.09)	0.13 (0.12, 0.15)	0.19 (0.18, 0.2)	0.47 (0.46, 0.48)	0.28 (0.28, 0.29)
	10 year	0.09 (0.06, 0.12)	0.16 (0.15, 0.17)	0.23 (0.22, 0.24)	0.52 (0.51, 0.53)	0.32 (0.31, 0.33)
Black (N = 9,581)	2 year	0.01 (0, 0.02)	0.05 (0.04, 0.06)	0.08 (0.07, 0.09)	0.23 (0.21, 0.25)	0.11 (0.11, 0.12)
	3 year	0.01 (0, 0.03)	0.07 (0.05, 0.08)	0.11 (0.1, 0.12)	0.28 (0.26, 0.3)	0.14 (0.13, 0.15)
	5 year	0.03 (0.01, 0.05)	0.09 (0.08, 0.11)	0.14 (0.13, 0.15)	0.36 (0.33, 0.38)	0.18 (0.17, 0.19)
	8 year	0.04 (0.01, 0.07)	0.14 (0.12, 0.16)	0.18 (0.16, 0.2)	0.42 (0.4, 0.45)	0.23 (0.22, 0.24)
	10 year	0.06 (0.02, 0.09)	0.17 (0.15, 0.19)	0.22 (0.2, 0.24)	0.47 (0.44, 0.5)	0.26 (0.25, 0.28)
Hispanic (N = 2,040)	2 year	0.05 (0, 0.15)	0.06 (0.03, 0.09)	0.07 (0.05, 0.09)	0.26 (0.23, 0.29)	0.17 (0.15, 0.18)
	3 year	0.05 (0, 0.15)	0.08 (0.04, 0.12)	0.09 (0.06, 0.11)	0.31 (0.27, 0.34)	0.19 (0.17, 0.21)
	5 year	0.05 (0, 0.15)	0.09 (0.05, 0.13)	0.12 (0.09, 0.14)	0.39 (0.35, 0.42)	0.24 (0.22, 0.27)
	8 year	0.13 (0, 0.29)	0.11 (0.06, 0.15)	0.19 (0.15, 0.23)	0.48 (0.43, 0.52)	0.32 (0.29, 0.34)
	10 year	0.13 (0, 0.29)	0.13 (0.08, 0.18)	0.24 (0.18, 0.29)	0.51 (0.46, 0.55)	0.35 (0.32, 0.38)
White (N = 23,872)	2 year	0.04 (0, 0.07)	0.07 (0.05, 0.08)	0.08 (0.08, 0.09)	0.26 (0.25, 0.27)	0.16 (0.15, 0.16)
	3 year	0.07 (0.02, 0.11)	0.07 (0.06, 0.09)	0.1 (0.1, 0.11)	0.31 (0.3, 0.32)	0.19 (0.18, 0.19)
	5 year	0.07 (0.03, 0.12)	0.1 (0.09, 0.11)	0.14 (0.13, 0.15)	0.39 (0.38, 0.4)	0.24 (0.23, 0.25)
	8 year	0.09 (0.04, 0.14)	0.13 (0.12, 0.15)	0.19 (0.18, 0.21)	0.48 (0.47, 0.49)	0.3 (0.29, 0.31)
	10 year	0.13 (0.07, 0.19)	0.15 (0.14, 0.17)	0.23 (0.22, 0.25)	0.53 (0.52, 0.55)	0.34 (0.33, 0.35)

© 2024 Stock SR et al. *JAMA Network Open.*

eTable 15. Cumulative incidence estimates of time from nmCRPC diagnosis to mCRPC disease progression or death as competing risks.

Estimates are provided among all patients, stratified by categories of race/ethnicity, stratified by categories of age, and stratified by combined categories of race/ethnicity and age.

		**Age**				
**Transition**	**Cumulative Incidence Estimate**	< 55	55-64	65-74	≥75	**All Patients**
mCRPC						
All Patients (N = 8,518)	2 year	0.46 (0.31, 0.58)	0.53 (0.49, 0.56)	0.5 (0.48, 0.52)	0.41 (0.39, 0.42)	0.45 (0.44, 0.47)
	3 year	0.5 (0.35, 0.62)	0.61 (0.57, 0.64)	0.59 (0.57, 0.61)	0.47 (0.45, 0.48)	0.52 (0.51, 0.53)
	5 year	0.58 (0.42, 0.69)	0.7 (0.66, 0.73)	0.67 (0.65, 0.69)	0.54 (0.52, 0.55)	0.6 (0.59, 0.61)
	8 year	0.68 (0.49, 0.8)	0.76 (0.73, 0.79)	0.74 (0.72, 0.75)	0.58 (0.57, 0.6)	0.65 (0.64, 0.66)
	10 year	0.68 (0.49, 0.8)	0.79 (0.75, 0.82)	0.76 (0.74, 0.78)	0.59 (0.58, 0.61)	0.67 (0.65, 0.68)
Black (N = 2,313)	2 year	0.46 (0.27, 0.6)	0.44 (0.38, 0.49)	0.44 (0.4, 0.48)	0.37 (0.34, 0.4)	0.41 (0.39, 0.43)
	3 year	0.49 (0.29, 0.63)	0.51 (0.45, 0.57)	0.52 (0.49, 0.56)	0.43 (0.39, 0.46)	0.48 (0.46, 0.5)
	5 year	0.58 (0.36, 0.72)	0.59 (0.53, 0.65)	0.61 (0.58, 0.65)	0.51 (0.47, 0.54)	0.56 (0.54, 0.58)
	8 year	0.58 (0.36, 0.72)	0.69 (0.63, 0.74)	0.7 (0.66, 0.74)	0.57 (0.54, 0.6)	0.63 (0.61, 0.66)
	10 year	0.58 (0.36, 0.72)	0.73 (0.67, 0.78)	0.74 (0.7, 0.78)	0.59 (0.56, 0.62)	0.66 (0.64, 0.69)
Hispanic (N = 531)	2 year	0.33 (0, 0.7)	0.52 (0.31, 0.67)	0.47 (0.38, 0.55)	0.32 (0.26, 0.37)	0.38 (0.34, 0.43)
	3 year	0.33 (0, 0.7)	0.59 (0.37, 0.74)	0.58 (0.48, 0.66)	0.39 (0.33, 0.44)	0.46 (0.41, 0.51)
	5 year	0.330 (0, 0.7)	0.63 (0.4, 0.77)	0.68 (0.58, 0.76)	0.48 (0.42, 0.54)	0.55 (0.5, 0.59)
	8 year	0.58 (0, 0.89)	0.67 (0.45, 0.8)	0.79 (0.69, 0.85)	0.58 (0.52, 0.63)	0.65 (0.6, 0.69)
	10 year	0.58 (0, 0.89)	0.73 (0.48, 0.86)	0.8 (0.7, 0.86)	0.6 (0.54, 0.65)	0.66 (0.62, 0.7)
White (N = 5,344)	2 year	0.5 (0.12, 0.72)	0.61 (0.55, 0.65)	0.53 (0.51, 0.56)	0.43 (0.41, 0.45)	0.48 (0.46, 0.49)
	3 year	0.58 (0.18, 0.79)	0.69 (0.63, 0.73)	0.61 (0.59, 0.64)	0.49 (0.47, 0.51)	0.55 (0.53, 0.56)
	5 year	0.72 (0.21, 0.9)	0.79 (0.74, 0.83)	0.7 (0.67, 0.72)	0.55 (0.53, 0.57)	0.62 (0.6, 0.63)
	8 year	0.81 (0.22, 0.96)	0.83 (0.78, 0.87)	0.75 (0.72, 0.77)	0.58 (0.57, 0.6)	0.66 (0.64, 0.67)
	10 year	0.81 (0.22, 0.96)	0.84 (0.79, 0.88)	0.76 (0.74, 0.78)	0.59 (0.57, 0.61)	0.66 (0.65, 0.68)

© 2024 Stock SR et al. *JAMA Network Open.*

		Age				
**Transition**	**Cumulative Incidence Estimate**	< 55	55-64	65-74	≥75	**All Patients**
Death						
All Patients (N = 8,518)	2 year	0.02 (0, 0.05)	0.04 (0.02, 0.05)	0.05 (0.04, 0.06)	0.17 (0.16, 0.18)	0.11 (0.11, 0.12)
	3 year	0.02 (0, 0.05)	0.05 (0.03, 0.07)	0.06 (0.05, 0.07)	0.23 (0.22, 0.24)	0.15 (0.14, 0.16)
	5 year	0.02 (0, 0.05)	0.06 (0.05, 0.08)	0.09 (0.08, 0.1)	0.31 (0.3, 0.32)	0.21 (0.2, 0.22)
	8 year	0.05 (0, 0.11)	0.08 (0.06, 0.1)	0.1 (0.09, 0.12)	0.38 (0.36, 0.39)	0.25 (0.24, 0.26)
	10 year	0.05 (0, 0.11)	0.09 (0.06, 0.11)	0.12 (0.1, 0.13)	0.4 (0.38, 0.42)	0.27 (0.26, 0.28)
Black (N = 2,313)	2 year	0 (0, 0)	0.05 (0.03, 0.08)	0.05 (0.03, 0.07)	0.15 (0.13, 0.18)	0.09 (0.08, 0.11)
	3 year	0 (0, 0)	0.06 (0.04, 0.09)	0.06 (0.04, 0.08)	0.21 (0.18, 0.24)	0.13 (0.11, 0.14)
	5 year	0 (0, 0)	0.08 (0.05, 0.11)	0.1 (0.07, 0.12)	0.29 (0.26, 0.32)	0.18 (0.16, 0.19)
	8 year	0.05 (0, 0.15)	0.11 (0.07, 0.14)	0.11 (0.09, 0.14)	0.37 (0.34, 0.4)	0.23 (0.21, 0.25)
	10 year	0.05 (0, 0.15)	0.12 (0.08, 0.16)	0.13 (0.1, 0.15)	0.41 (0.37, 0.44)	0.25 (0.23, 0.27)
Hispanic (N = 531)	2 year	0.33 (0, 0.7)	0.03 (0, 0.1)	0.02 (0, 0.05)	0.17 (0.12, 0.22)	0.12 (0.08, 0.15)
	3 year	0.33 (0, 0.7)	0.03 (0, 0.1)	0.02 (0, 0.05)	0.23 (0.18, 0.28)	0.15 (0.12, 0.19)
	5 year	0.33 (0, 0.7)	0.08 (0, 0.18)	0.02 (0, 0.05)	0.31 (0.25, 0.36)	0.21 (0.17, 0.25)
	8 year	0.33 (0, 0.7)	0.08 (0, 0.18)	0.05 (0.01, 0.09)	0.4 (0.34, 0.46)	0.27 (0.23, 0.32)
	10 year	0.33 (0, 0.7)	0.08 (0, 0.18)	0.06 (0.01, 0.11)	0.42 (0.36, 0.48)	0.29 (0.25, 0.34)
White (N = 5,344)	2 year	0 (0, 0)	0.03 (0.01, 0.04)	0.05 (0.04, 0.06)	0.18 (0.17, 0.19)	0.12 (0.11, 0.13)
	3 year	0 (0, 0)	0.04 (0.02, 0.06)	0.07 (0.06, 0.08)	0.24 (0.22, 0.26)	0.17 (0.16, 0.18)
	5 year	0 (0, 0)	0.05 (0.02, 0.07)	0.09 (0.08, 0.11)	0.32 (0.3, 0.34)	0.22 (0.21, 0.23)
	8 year	0 (0, 0)	0.05 (0.03, 0.08)	0.11 (0.09, 0.12)	0.37 (0.36, 0.39)	0.26 (0.25, 0.27)
	10 year	0 (0, 0)	0.06 (0.03, 0.09)	0.12 (0.1, 0.13)	0.39 (0.38, 0.41)	0.28 (0.26, 0.29)

© 2024 Stock SR et al. *JAMA Network Open.*

eTable 16. Cumulative incidence estimates of time from mCRPC diagnosis to death.

Estimates are provided among all patients, stratified by categories of race/ethnicity, stratified by categories of age, and stratified by combined categories of race/ethnicity and age.

		Age				
**Transition**	**Cumulative Incidence Estimate**	< 55	55-64	65-74	≥75	**All Patients**
Death						
All Patients (N = 22,963)	2 year	0.31 (0.24, 0.37)	0.3 (0.29, 0.32)	0.3 (0.29, 0.31)	0.48 (0.47, 0.49)	0.39 (0.38, 0.4)
	3 year	0.44 (0.36, 0.51)	0.41 (0.39, 0.43)	0.42 (0.41, 0.43)	0.62 (0.61, 0.63)	0.52 (0.51, 0.52)
	5 year	0.54 (0.46, 0.61)	0.56 (0.54, 0.58)	0.59 (0.58, 0.61)	0.8 (0.79, 0.81)	0.68 (0.68, 0.69)
	8 year	0.64 (0.54, 0.71)	0.69 (0.66, 0.71)	0.72 (0.7, 0.74)	0.91 (0.89, 0.92)	0.8 (0.79, 0.81)
	10 year	0.65 (0.56, 0.73)	0.74 (0.71, 0.76)	0.77 (0.75, 0.79)	0.94 (0.92, 0.95)	0.84 (0.83, 0.85)
Black (N = 5,592)	2 year	0.24 (0.15, 0.33)	0.26 (0.23, 0.29)	0.27 (0.25, 0.29)	0.44 (0.42, 0.47)	0.33 (0.32, 0.35)
	3 year	0.42 (0.3, 0.51)	0.36 (0.32, 0.39)	0.39 (0.37, 0.42)	0.58 (0.55, 0.6)	0.45 (0.44, 0.47)
	5 year	0.55 (0.42, 0.65)	0.48 (0.44, 0.51)	0.54 (0.52, 0.57)	0.77 (0.74, 0.79)	0.61 (0.6, 0.63)
	8 year	0.67 (0.53, 0.77)	0.63 (0.58, 0.67)	0.68 (0.65, 0.72)	0.89 (0.86, 0.91)	0.75 (0.73, 0.77)
	10 year	0.67 (0.53, 0.77)	0.67 (0.62, 0.72)	0.76 (0.71, 0.8)	0.94 (0.91, 0.96)	0.8 (0.77, 0.82)
Hispanic (N = 1,143)	2 year	0.36 (0, 0.66)	0.26 (0.17, 0.33)	0.26 (0.2, 0.31)	0.42 (0.37, 0.46)	0.35 (0.31, 0.38)
	3 year	0.36 (0, 0.66)	0.34 (0.24, 0.43)	0.36 (0.3, 0.42)	0.54 (0.49, 0.58)	0.45 (0.42, 0.49)
	5 year	0.36 (0, 0.66)	0.54 (0.42, 0.64)	0.48 (0.41, 0.54)	0.74 (0.68, 0.78)	0.62 (0.58, 0.66)
	8 year	0.36 (0, 0.66)	0.7 (0.55, 0.8)	0.61 (0.52, 0.68)	0.83 (0.77, 0.88)	0.74 (0.69, 0.78)
	10 year	NA*	0.76 (0.6, 0.86)	0.64 (0.53, 0.73)	0.85 (0.79, 0.9)	0.77 (0.72, 0.81)
White (N = 15,517)	2 year	0.4 (0.29, 0.5)	0.34 (0.32, 0.37)	0.32 (0.3, 0.33)	0.5 (0.49, 0.51)	0.41 (0.41, 0.42)
	3 year	0.5 (0.38, 0.59)	0.45 (0.42, 0.48)	0.43 (0.42, 0.45)	0.64 (0.63, 0.66)	0.54 (0.54, 0.55)
	5 year	0.58 (0.45, 0.68)	0.62 (0.59, 0.66)	0.62 (0.6, 0.63)	0.81 (0.8, 0.82)	0.72 (0.71, 0.73)
	8 year	0.63 (0.49, 0.73)	0.73 (0.7, 0.76)	0.75 (0.72, 0.77)	0.92 (0.91, 0.93)	0.83 (0.82, 0.84)
	10 year	0.67 (0.51, 0.77)	0.78 (0.75, 0.82)	0.78 (0.76, 0.81)	0.94 (0.93, 0.96)	0.86 (0.85, 0.87)

*unable to estimate

© 2024 Stock SR et al. *JAMA Network Open.*
