# Supplementary material for: Racial and Ethnic Differences in Prostate Cancer Epidemiology Across Disease States in the VA
Source: JAMA Netw Open. 2024 Nov 15;7(11):e2445505. doi: 10.1001/jamanetworkopen.2024.45505 (PMC11568464; doi:10.1001/jamanetworkopen.2024.45505)
Supplement: Supplement 2. — Data Sharing Statement [file jamanetwopen-e2445505-s002.pdf]

Data Sharing Statement

Stock. Racial and Ethnic Differences in Prostate Cancer Epidemiology Across Disease States in the VA. *JAMA Netw Open*. Published November 15, 2024.
doi:10.1001/jamanetworkopen.2024.45505

Data
Data available: No

Additional Information
Explanation for why data not available: The data includes personal health information
